# RNA-Seq reveals complex genetic response to deepwater horizon oil release in *Fundulus grandis*

**DOI:** 10.1186/1471-2164-13-474

**Published:** 2012-09-12

**Authors:** Tzintzuni I Garcia, Yingjia Shen, Douglas Crawford, Marjorie F Oleksiak, Andrew Whitehead, Ronald B Walter

**Affiliations:** 1 Department of Chemistry and Biochemistry, Texas State University, 601 University Drive, San Marcos, TX 78666-4616, USA; 2 Rosenstiel School of Marine & Atmospheric Science, University of Miami, 4600 Rickenbacker Causeway, Miami, FL 33149, USA; 3 Department of Environmental Toxicology, University of California Davis, One Shields Avenue, Davis, CA 95616

**Keywords:** Digital gene expression, Toxicology, Annotation, Bioinformatics, De novo assembly, Non-model organism, Transcriptome

## Abstract

**Background:**

The release of oil resulting from the blowout of the Deepwater Horizon (DH) drilling platform was one of the largest in history discharging more than 189 million gallons of oil and subject to widespread application of oil dispersants. This event impacted a wide range of ecological habitats with a complex mix of pollutants whose biological impact is still not yet fully understood. To better understand the effects on a vertebrate genome, we studied gene expression in the salt marsh minnow *Fundulus grandis*, which is local to the northern coast of the Gulf of Mexico and is a sister species of the ecotoxicological model *Fundulus heteroclitus*. To assess genomic changes, we quantified mRNA expression using high throughput sequencing technologies (RNA-Seq) in *F. grandis* populations in the marshes and estuaries impacted by DH oil release. This application of RNA-Seq to a non-model, wild, and ecologically significant organism is an important evaluation of the technology to quickly assess similar events in the future.

**Results:**

Our *de novo* assembly of RNA-Seq data produced a large set of sequences which included many duplicates and fragments. In many cases several of these could be associated with a common reference sequence using blast to query a reference database. This reduced the set of significant genes to 1,070 down-regulated and 1,251 up-regulated genes. These genes indicate a broad and complex genomic response to DH oil exposure including the expected *AHR*-mediated response and CYP genes. In addition a response to hypoxic conditions and an immune response are also indicated. Several genes in the choriogenin family were down-regulated in the exposed group; a response that is consistent with AH exposure. These analyses are in agreement with oligonucleotide-based microarray analyses, and describe only a subset of significant genes with aberrant regulation in the exposed set.

**Conclusion:**

RNA-Seq may be successfully applied to feral and extremely polymorphic organisms that do not have an underlying genome sequence assembly to address timely environmental problems. Additionally, the observed changes in a large set of transcript expression levels are indicative of a complex response to the varied petroleum components to which the fish were exposed.

## Background

In April of 2010, the explosion of the Deepwater Horizon oil drilling platform initiated the largest deep water oil release in the history of the petroleum industry culminating in more than 189 million gallons of crude oil released into the environment [1,2]. This incident has greatly impacted the natural and economic resources of many Gulf of Mexico coastal areas [3-7].

For several decades *Fundulus heteroclitus* has been an important model organism employed in environmental toxicology studies of heavily polluted superfund sites throughout the northeastern United States [8]. We have leveraged the body of existing scientific work surrounding *F. heteroclitus* in the analysis of high throughput sequencing techniques applied to natural *Fundulus grandis* populations, a closely-related sister species inhabiting DH-impacted sites in the Gulf of Mexico [9]. This application holds promise for revealing induction of environmental stress with genome-wide molecular genetic response patterns. Deploying these techniques within natural populations for which no underlying genomic information is available also holds the potential to track responses in the most situation-appropriate and ecologically relevant species, and be used to follow ecosystem recovery [10]. Herein we provide methodological detail and results of transcriptome expression comparisons between populations of *F. grandis* sampled directly from the environment affected by the DH oil release versus those from unaffected populations.

In this study we assembled a reference transcriptome from representative natural populations of *F. grandis* collected from Gulf Coast estuaries ranging from Texas to Florida. We quantified liver mRNA expression from sequences produced using the Illumina GAIIx platform. A statistical framework was used to identify genes that were differentially expressed in oil-exposed versus non-exposed samples. Our results show a complex response including activation of AHR and related pathways previously shown to be important in response to xenobiotic hydrocarbons, as well as some previously undocumented gene responses. We also show that these results compare favorably with a microarray-based method. Additionally we compiled abundant transcript sequence data for a species not previously studied in this manner. And finally, we demonstrate the efficacy of these techniques for addressing similar events in the future while studying organisms key to a habitat instead of being restricted to a well studied model of lesser significance.

## Results and discussion

### Short read filtration

The exposed (*E*) samples *E1*, *E2* and *E3* and the unexposed (*U*) *U1* and *U2* samples had between 81% and 94% overall pass rates and most of the reads that passed remained paired (Table 1). After filtration 409,153,209 reads remained.

**Table 1:** Sample information

	**Unexposed(*U*)**	**Exposed Individuals(*E*)**			
	***U1***	***U2***	***E1***	***E2***	***E3***
Location*	1-3	5-8	4	4	4
Prior Hypoxia†	locations 1,3	location 6	no data	no data	no data
Collection Date	April 2008	April 2008	June 28, 2010	June 28, 2010	June 28, 2010
Individuals in Sample	6	8	1	1	1
Read Pairs	25,757,552	37,299,468	26,416,910	22,400,861	22,920,486
Single Reads	12,856,844	3,849,038	7,802,114	7,017,190	5,451,850
Aligned Fragments	2,919,635	4,907,552	4,722,098	3,844,805	4,180,692

*Locations: 1) Port Aransas, *TX* [27°45'59" N, 97°7'33" W], 2) Cocodrie, *LA* [29°15'13.89" N, 90°39'45.91" W], 3) Leeville, *LA* [29°12'43.37" N, 90°09'08.37" W], 4) Isle Grande Terre, *LA* [29°16'22.93" N, 89°56'41.87" W], 5) Dauphin Island, *AL* [30°20'04.92" N, 88°07'57.21" W], 6) Weeks Bay, *AL* [30°22'41.80" N, 87°50'19.60" W], 7) Santa Rosa Island, *FL* [30°21'16.63" N, 87°2'46.92" W], 8) Florida State University Marine Station, *FL* [29°54'56.83" N, 84°30'39.07" W]. †History further detailed in Additional file 1: Table S3.

### Transcript assembly

We selected a transcriptome assembly based on the features of the N50 plot over a range of k-mer lengths from 21 to 49. The point k = 27 was the highest k-mer length in what seemed to be a plateau of N50 values before the N50 began to dip (Additional file 1: Figure S1). The average length over all sequences in this assembly is 599bp with an N50 value of 1,238bp, and when contigs shorter than 500bp are excluded these values rise to 1,429bp and 1,804bp respectively. These statistics indicate a population of robust and contiguous sequences among smaller fragmentary contigs. The final set obtained after mapping reads and removing those transcripts with fewer than 10 mapped reads contains 120,725 contigs with an average length of 878 bp and an N50 of 1,494 bp. We were able to annotate 45% of the assembled contigs with a total of 15,494 unique sequence descriptions.

While this set still contains many small fragments and some sequence overlaps, it indicates that two sequencing technologies are not necessary for *de novo* assembly of non-model organisms as is common practice in recently published studies [11,12] with which our methods compare favorably.

### Differentially expressed genes

**Figure 1:**
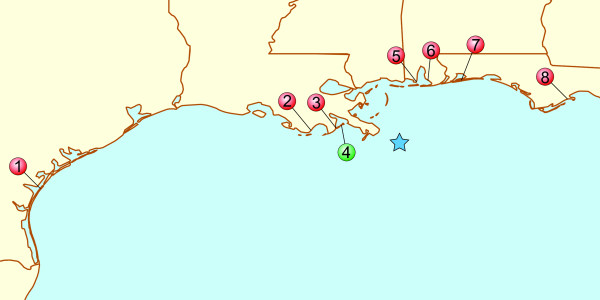
**Sample collection map.** The sample collection sites are shown here on a map of the northern coast of the Gulf of Mexico. Sample sites 1-3 and 5-8 are pooled into the unexposed reference samples 1 and 2 respectively and were collected during April of 2008. Samples from site 4 were taken on June 28, 2010, when the oil had been present at the location for at least 2 weeks. The blue star indicates the position of the Deepwater Horizon blowout which occurred on April 20, 2010 and was effectively uncontrolled until July 15, 2010, though the well was not officially sealed until September 19, 2010.

**Figure 2:**
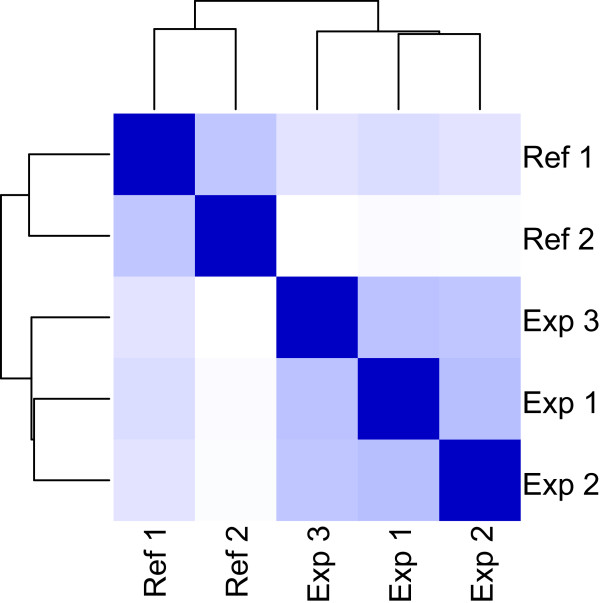
**Sample Clustering.** Samples are clustered by similarity of gene expression values. The blocks in the comparison matrix are scaled by color so that the most similar are dark blue and least similar are white. The samples from each treatment cluster together indicating global differences between the two treatments.

There exists subtle but significant genetic structure within *F. grandis*, where population divergence generally follows a pattern of isolation-by-distance with longitude across the northern Gulf of Mexico. Our “western” pool of samples (sites 1-3 on Figure 1) included fish from the western phylogeographic cluster of populations, whereas the “eastern” pool of samples (sites 5-8 on Figure 1) included fish from the eastern phylogeographic cluster of populations [13]. Grand Terre, our oil-impacted site, is located near the center of the species’ range (site 4 on Figure 1), and fish from this site have greatest affinity to the western phylogeographic cluster of populations. Among our reference fish are animals from Leeville, LA, which is only 21.26 km from the Grand Terre site; significant genetic structure is not observed within this species at that geographic scale [13]. Since our reference samples are representative of the genetic diversity within the species, including from southern Louisiana, we consider it unlikely that genetic variation among populations confounds the treatment effects that we observe. Seasonal changes should also be minimal as fish were collected during the months of April (reference) and June (exposed). As shown in Figure 2, the unexposed samples cluster separately from the exposed samples bearing out the self-similarity of the pooled unexposed samples, and indicating global differences between the two sets. 

**Figure 3:**
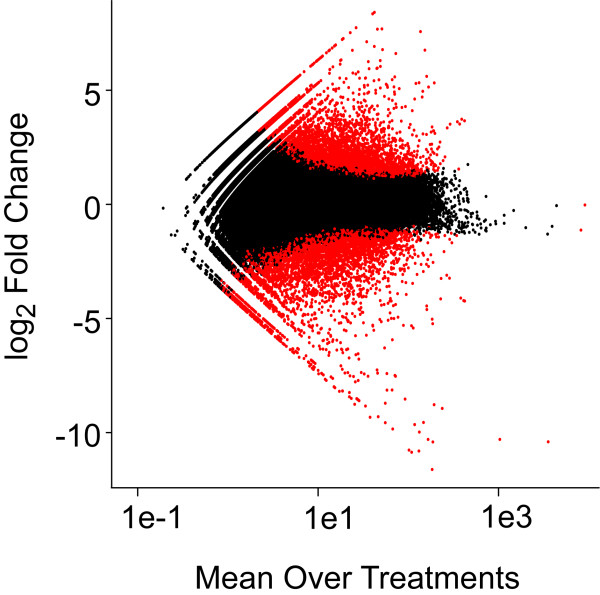
**Significant differences found in oil exposed samples *vs. *unexposed samples.** Each point represents the mean expression level plotted against the fold change for a given sequence. Black points are not statistically significant, and red points are significant at P < 0.01.

**Figure 4:**
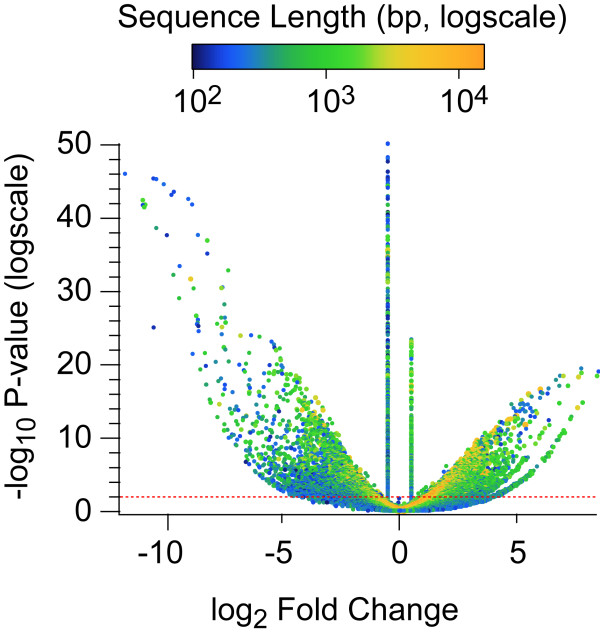
**Volcano plot of the oil exposed (*E*) versus the unexposed (*U*) treatments.** The horizontal axis is the log_2_ fold change between the treatment means. The -log_10_ (P-value) is plotted on the vertical axis. Each sequence is represented by one point on the graph. The length of each transcript is indicated by the color of the point in reference to the color scale at the top. The transition from blue to green occurs at roughly 300bp, and from green to orange occurs at roughly 3000bp. This creates three categories with 32,527 sequences shorter than 300bp, then 83,043 sequences between 300 and 3,000bp, and finally 5,155 sequences over 3,000bp. With this many points, some occlusion is unavoidable but in general points representing longer sequences provide more statistical power than shorter sequences. Points above the dotted red line indicate sequences that pass the P < 0.01 significance threshold. The two vertical columns of points in the chart are sequences for which expression was only detected in one treatment so the log_2_ fold change values were artificially set to (±) 0.5 (instead of +/- infinity) as appropriate.

At a significance level of P < 0.01, we determined a set of 5,384 up and 5,882 down-regulated sequences (Figures 3 and 4). The assembly contains long well-assembled sequences in addition to short fragments, and the color-coding of points by length reveals that the longer sequences tend to provide more statistical power (green to orange points in Figure 4). The short length of these reads, however, is beneficial when matching them to short sequence fragments. This allows many of the shorter contigs to also provide useful data (blue points in Figure 4). Many of the sequences are alternative assemblies of the same original transcript or are fragments of the same transcript. Thus, we used the annotations derived from blast searching against the non-redundant database (nr) to group transcripts that likely represent the same gene by unique sequence annotations. This resulted in 1,070 down-regulated and 1,251 up-regulated unique sequence annotations. That still leaves 6,146 significantly differently expressed sequences that lack annotation, where 962 of these sequences are over 1kb in length. Some of these may be entirely new gene products unknown to prior studies while others are likely assembly errors.

Among the more interesting genes that were differentially expressed are two up-regulated aryl-hydrocarbon receptor sequences (*AHR1B* and *AHR2*) (Figure 3). The response of *AHR* pathway genes has been well documented following exposure to aromatic hydrocarbons, where activation of this pathway is diagnostic of exposure to this class of pollutant [8,14,15]. Downstream in the AHR response pathway are cytochrome P450 genes; *CYP1C* (mediated by *AHR2*) and *CYP1B1* are found to be up-regulated in the exposed sample (Figure 3), while others in the *CYP2N* and *CYP2X* sub-families are down-regulated (Additional file 1: Table S2). Whereas previous studies have highlighted the response of *CYP1A* to dioxins, polycyclic aromatic hydrocarbons (PAHs), and other similar pollutants, in this analysis we detected no significant difference in *CYP1A* mRNA expression upon exposure to DH oil. To verify this we searched the assembled *F. grandis* transcript sequences using the *F. heteroclitus CYP1A* sequence and found that only small fragments existed in the *de novo* assembly. We then mapped *F. grandis* reads to the *F. heteroclitus CYP1A* sequence and analyzed its expression in the context of the assembled sequences. While there was an increase in the expression values in the oil exposed sample relative to either of the unexposed samples, it did not pass the p-value threshold. It may be that by the time of collection the animals had been exposed to levels and durations of DH oil or some prior exposure causing a de-sensitized response by *CYP1A* response in the exposed population. Alternately, since a previous study that used the same RNA-Seq data did find a significant increase in *CYP1A*, but used *F. heteroclitus* ESTs as the reference transcriptome [16], it could be that the residual fragmentary and erroneous sequences in the *de novo* assembly led to reduced statistical power for some sequences.

**Figure 5:**
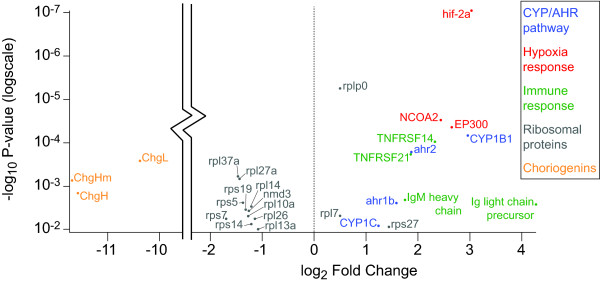
**Detail of selected gene expression.** This is a selection of sequences from the larger volcano plot in Figure 2. Each point is labeled with a gene name or sequence description if gene name was unavailable. The genes are grouped into five general groups illustrating a particular response as indicated by color. Note that a large section of empty space has been removed from the horizontal range for legibility.

Given clear damage to killifish gills caused by contaminating oil [16], one might anticipate that compromised gill function, coupled with limited oxygen exchange between air and water in oil-slick contaminated areas, could result in hypoxic stress. In the up-regulated gene set from DH oil exposed animals we find several genes suggesting a hypoxic response. *Hypoxia inducible factor 2-alpha*, its co-activator *E1A binding protein p300*, and the activity enhancing *nuclear receptor coactivator 2* are all up-regulated [17] (Figure 5). It should be noted that the estuaries inhabited by *F. grandis* often experience transient periods of hypoxia (see Additional file 1: Table S2), and given the broad range of sampling sites it is likely that our reference set includes RNA from individuals experiencing hypoxic conditions to some degree, thus capturing a range of normal and hypoxic expression levels for these genes. The inclusion of reference individuals exposed to hypoxia would make it more difficult to statistically identify a hypoxic response in the exposed population. Therefore, the genes indicated here are likely a conservative set of hypoxia response genes. 

Another broad set of up-regulated genes in DH oil exposed fish indicates an immune response perhaps by circulatory leukocytes. The secreted form of the immunoglobulin mu heavy chain and heavy chain variable regions are both indicative of an active immune response. Tumor necrosis factor receptors 14 and 21, involved in immune response signaling pathways, are both up-regulated (Figure 5). Previous studies have noted an association between an immune response induced by hypoxia and/or *HIF1* activation [18], and hydrocarbons are well-known to alter immune system function [19].

The set of genes that appears to be down-regulated in response to DH oil exposure is also large and diverse. One large group of down regulated transcripts includes a wide selection of 40S and 60S ribosomal proteins (Figure 5). A similar response has been reported in hypoxic conditions in zebrafish gills [20], and may be explained by an overall reduction in protein synthesis as a survival strategy during hypoxic conditions [21].

One final group of genes that was previously observed to be strongly down-regulated are the group of choriogenins *ChgL*, *ChgH*, and *ChgHm*[16]. The sequences for these are highly repetitive and so did not assemble well. Thus they did not originally show up in our analysis except as unidentified small fragments albeit with expression levels that would confirm earlier observations. To better examine the expression levels in the context of the rest of the assembly, we added the *F. heteroclitus* cDNA sequences to the assembled contigs and recalculated differential expression for these in the context of the rest of the assembled sequences. The results confirm that choriogenin genes are strongly down-regulated (Figure 5). As has been previously noted, the expression of these genes is estrogen-dependent and is down-regulated by exposure to PAHs [22].

### Comparison to microarrays

**Figure 6:**
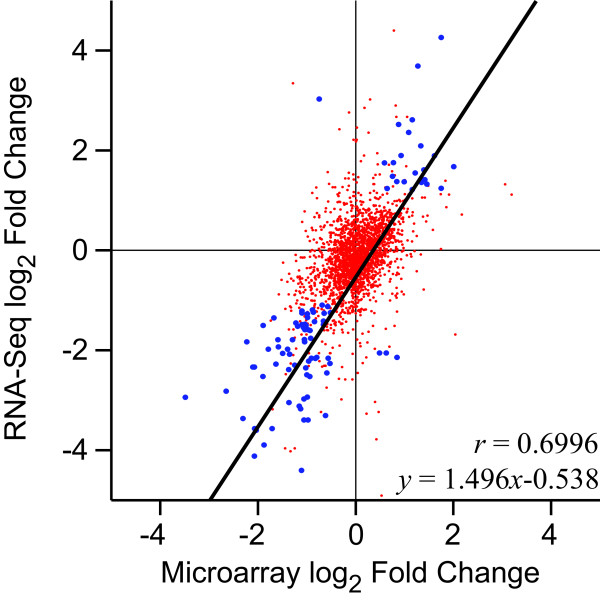
**Comparison of expression changes in RNA-Seq and microarray-based methods.** The log_2_ fold-change in expression values measured by the RNA-Seq based method presented here are plotted against those measured by microarray analysis (microarray data from [16]). The red spots are representative of the full set of 2,290 sequences from the microarray for which a best hit was selected from the *de novo* assembly. The blue spots indicate sequences that were statistically significant in both comparisons to a threshold of p < 0.01. Three sequences are not shown that were down-regulated in the microarray analysis but seemed to be turned off completely in the RNA-Seq analysis as a result of exposure. Another four sequences are not shown which would have expanded the scale greatly but for which both techniques agreed in the direction of regulation if not the magnitude. A linear model is fit to the blue points, and the Pearson’s correlation is given as well.

The results of the microarray-based analysis correlate well with those measured by RNA-Seq (Figure 6). One notable difference is that the dynamic range of expression levels seems to be much wider in the RNA-Seq method versus those observed for microarray results. The RNA-Seq method exhibits results for values ranging from approximately +5 to -14 log_2_ fold change while the microarray results are in a more narrow range of approximately ± 3.5fold. This may indicate a greater sensitivity in the RNA-Seq based methodology which is an observation that is consistent with other similar comparisons [23]. For the set of best hits between the data sets we calculated a Pearson’s correlation of *r* = 0.48, while the set of sequences found to be significant in both analyses had an *r* value of 0.70. These indicate a reasonable correlation between the two data sets with a high degree of variability, and are similar to results of other such comparisons [23]. Much of the variation between the data sets is likely related to the fact that only the exposed samples were common among the two analyses; the un-exposed samples in each used mRNA from different sets of fish. The unexposed RNA-Seq data represent two pools of 6 and 8 individuals collected from sites on both sides of the Mississippi river, while the microarray data set is comprised of a separate set of 75 individual animals collected in the summer of 2010 from different sites along the Gulf of Mexico. 

## Conclusions

Assembly of mRNA transcript data is a challenging task that is complicated further when using wild outbred animals that have significant genetic variability between and within populations and which lack prior EST or genome sequence data. However, data provided here suggest that useful analysis can be achieved with reference to phylogenetically similar species. Additionally, Illumina short read data have recently been shown to be more resilient to fragmentary, incomplete sequences as targets for alignment for digital gene expression [11] as would likely be the case in non-model organisms. We suggest that with improvements in assembly techniques, digital gene expression results are likely to improve and become increasingly valuable tools for assessing environmental damage and recovery after anthropogenic perturbation. Thus, the methods provided here indicate that RNA-Seq can be used to investigate global mRNA expression levels in a very wide variety of organisms, such that the most appropriate species for particular research questions in environmental science may be exploited.

In this case, RNA-Seq studies of wild populations of the small teleost fish (*Fundulus grandis)* collected from estuaries in the Gulf of Mexico show broad and complex molecular genomic responses to DH oil exposure. In comparison to samples collected from a varied set of Gulf of Mexico coastal sites in 2008, the fish from estuaries with oil in the water exhibited mRNA expression patterns consistent with exposure to the toxic components of oil (aromatic hydrocarbons). In addition to expected indicators of AH exposure, the RNA-Seq based techniques used here revealed effects on several cellular systems, such as the immune response and effects on the wide variety of ribosomal proteins, that might not have been specifically interrogated by a more focused examination of specific pathways through smaller-scale techniques. Additionally, the specific gene responses we have analyzed and discussed here represent only a handful of the thousands of significant up and down regulated gene sets. This data set will continue to provide insight into the full effects of this oil exposure as results are compared with other similar studies. The RNA-Seq results are also similar to a distinct and independent microarray study and thus lend validity to the overall methods employed and results of the study.

## Methods

### Data collection

Samples of *Fundulus grandis* were collected from eight estuaries over a range of time points prior to, and concurrent with, the Deepwater Horizon oil release event. Collection sites, from west to east, include: 1) Port Aransas, TX [27°45'59"N, 97°7'33"W]; 2) Cocodrie, LA [29°15' 13.89" N, 90°39' 45.91" W]; 3) Leeville, LA [29° 12' 43.37N, 90° 09' 08.37" W]; 4) Isle Grande Terre, LA [29°16'22.93"N, 89°56'41.87"W]; 5) Dauphin Island, AL [30°20' 04.92"N, 88° 07'57.21"W]; 6) Weeks Bay, AL [30°22'41.80"N, 87°50'19.60"W]; 7) Santa Rosa Island, FL [30°21'16.63"N, 87° 2' 46.92"W]; and 8) Florida State University Marine Station, FL [29°54'56.83"N, 84°30'39.07"W]. Three adults were sampled from Isle Grande Terre (Barataria Bay, LA) on June 28, 2010 -- a time when oil had already heavily infiltrated the area for at least two weeks [16] (*exposed* samples *E1*, *E2*, *E3*). Each of the three individuals discussed thus far were kept as separate biological replicate samples for sequencing. Two additional samples were composed of 6 and 8 adults pooled from 7 sites East or West of the Mississippi (locations1-3 and 5-8) collected prior to the oil spill (April 2008) (*unexposed* samples *U1* and *U2* respectively) (Table 1). Both genders are represented in both exposed and unexposed samples, and differences in size are not expected to be a major factor in the current analysis [24-26]. In all cases the RNA was purified from isolated livers. Several of these sites have a recorded history of hypoxic events as indicated in Additional file 1: Table S2. None of the collection sites are in heavily industrialized areas with known histories of pollution. While small oil seeps occur throughout the Gulf of Mexico, we do not expect any to be exposed at the same levels as the south Louisiana fish during June 2010.

### RNA isolation

Oil exposed samples

RNA from adult male GT fish was isolated as reported in [16]. Briefly, liver tissues were dissected from fish field sites immediately following capture and preserved in RNAlater (Ambion, Austin, TX, USA). Total RNA was extracted using TRIzol reagent (Invitrogen, Carlsbad, CA, USA), purified using RNeasy spin columns (Qiagen Inc., Valencia, CA, USA), and RNA quality was verified using microfluidic electrophoresis (Experion instrument and reagents, Bio-Rad Laboratories, Hercules, CA, USA).

Control samples

RNA was isolated from livers stored in RNALater (20°C) by homogenizing tissue using ceramic beads homogenizer (Bio101, Savant; Carlsbad, CA). Tissues were place in screw-top 2ml tube with 750 ul of chaotropic reagent, 1.25 g ceramic beads and violently shaken for 40 s. Solution was centrifuged and 500 ul of supernatant placed in RNAse-free microfuge tube, with 50 ul 2M NaOAc pH 4.0, vortexed; 500ul of acidic phenol was added and vortexed again; then 100 ul of Chloroform:isoamyl alcohol (24:1) was added and vortexed. This solution was centrifuged at 4°C for 20 min. The upper-phase was placed in RNAse-free microfuge tube and RNA was precipitated with equal volume of isopropanol. RNA pellet was resuspended in chatropic reagent and purified using Qiagen RNA columns following manufacture’s procedures.

The quality of all mRNA samples were verified by electrophoresis on BioAnalyzer (Agilent, Santa Clara CA). The RIN scores for all samples were in the range of 8.3-9.7 (Additional file 1: Table S4).

### Short read processing

All RNA samples were sequenced in separate lanes on an Illumina Genome Analyzer (Expression Analysis, Inc. Durahm, NC). The resulting raw data files contained between 60-75 million 60 bp read pairs each for a total of 501,847,804 overall reads (deposited to NCBI short read archive, accession: SRA053469). We filtered and trimmed the reads based on quality scores using an in-house filtration algorithm that removed low scoring sections of each read and preserved the longest remaining fragment based on the following method. First, any reads with uncalled bases were rejected. The phred quality score of 2 encoded in fastq format as a ‘B’ is a special flag indicating that the results downstream of that position are untrustworthy. Therefore, as a second step, portions downstream of ‘B’ quality scores were removed. Finally, reads were broken apart anywhere the quality score value was 10 or below or where the average score of a position and its two neighbors was 20 or below. The largest remaining fragment of each read was kept (provided it was sufficiently long i.e., 49 bp or more) and the rest were discarded. Finally reads that lost their mate pair were moved into a single-end file and the integrity of the remaining read-pairing information was maintained. Any of the Perl scripts we developed to perform these steps are available on request.

### Reference transcriptome sequence assembly

We used the Velvet short read assembly package to perform the transcript assembly and made use of the Columbus and Oases extensions to Velvet [27,28] (Velvet version 1.1.04 and Oases version 0.1.21). As a first step we aligned the filtered and trimmed short reads to *F. heteroclitus* (a sister species to *F. grandis*) ESTs (retrieved from GenBank Feb 17, 2011) using the Bowtie short read aligner [29] (Bowtie version 0.12.7). The resulting alignment file (in SAM format), consisting of the read sequences and aligned locations if available, was then passed to Velvet along with the reference ESTs. We scanned a range of k-mer sizes from 21 to 49, and for each size we produced transcripts using Oases. Based primarily on the N50 value profile we selected the k = 27 assembly for further use in these studies (Figure S1). This full set of assembled sequences have been deposited to the Transcriptome Shotgun Assembly (TSA) database at GenBank (accession numbers JW540070 - JW662113) and are also available at xiphophorus.org under ‘Publication Supplements’.

We used Bowtie to map the full set of filtered reads to 203,252 assembled transcriptome sequences, then determined the number of reads mapped to each transcript. The settings we used allowed any read to map with up to 2 mismatches, and reads that could match in more than one location were randomly assigned. Any transcript with fewer than 10 mapped reads was rejected. This process eliminated 41% of the full transcript set resulting in a set of 120,725 transcripts that were used for further analyses.

### Analysis of differential expression

We again employed the Bowtie short read alignment program to map each sample of short reads independently to the refined set of reference transcripts. A custom Perl script then determined the number of reads mapped to each contig from the alignment file. A second Perl script then compiled the number of reads per contig per sample into a tabular format. The first column of the data file contains the transcript identifier, and each subsequent column has the number of fragments mapped to that transcript in each sample. We then used the DESeq package (ver 1.4.1, Bioconductor ver. 2.8, R ver. 2.13) to determine differential expression from the compiled tabular data [30]. DESeq uses a model based on the negative binomial distribution to determine significance and was developed specifically to meet the challenges of working with RNA-Seq data. The biological replicates were advantageous because DESeq is then able to determine a much better variance estimate for each transcript improving the statistical power of the experiment. The diagnostic plots produced by DESeq indicated no major problems with the data (Additional file 1: Figures S2, S3, and S4). A significance level of P < 0.01 was used to select differentially expressed sequences. Although there was a newer version of DESeq at the time, the one we employed was stable and used methods which had been published in the paper cited whereas the latest version was being updated daily in the Bioconductor repository.

### Sequence annotation

Matches for the reduced set of transcripts are sought from the non-redundant ‘nr’ blast database maintained by NCBI. We obtained the fasta format of the nr database on May 12, 2011, and prepared it for use with MPI-BLAST [31]. We used MPI-BLAST (ver 1.4.0) to query the *F. grandis* transcripts against nr and kept only 10 hits per sequence with an expect value threshold of 1e-10 using blastx. The results were processed by Blast2GO [32-34] in order to quickly assign a likely identity to each sequence.

### Comparison to microarray data

Microarray target sequences were queried against the assembled transcript sequences using blastn with a minimum expect-value threshold of 1e-10. For each microarray target, the hit with the highest bit score was selected as a match. RNA-Seq analyses were then compared to microarray data for *F. grandis* that compared samples from the exposed collection site (Isle Grande Terre, LA) both before and after the arrival of contaminating oil at that site, and compared the exposed site response to five different unexposed populations [16]. The microarray data were analyzed by mixed-model ANOVA which compared the response pre-oil to post-oil (3 timepoints) and between exposed and unexposed populations (6 populations) using JMP-SAS.

## Abbreviations

DH: Deepwater Horizon drilling platform; AHR: Aromatic hydrocarbon receptor; RNA: Seq - RNA sequencing; PAH: Polycyclic aromatic hydrocarbon.

## Competing interests

The authors declare that they have no competing interests.

## Authors’ contributions

TG carried out the assembly, digital gene expression, comparison to microarray results, analyzed results, and drafted the manuscript. YS consulted on design of the computational work and analysis, and contributed intellectually and critically to the manuscript. DC, MO, AW collected samples, conceived of the study, and contributed intellectually and critically to the manuscript. AW additionally carried out microarray analysis. RW conceived of the study, coordinated research, and helped to draft the manuscript. All authors read and approved the final manuscript.

## Supplementary Material

Additional file 1 **Figure S1.** Length/quantity statistics of assemblies for odd values of K from 21 to 49. In each graph that has two plots, the blue describes the full set of contigs while the red describes only those contigs 500bp or over. **Figure S2.** Plot of squared coefficient of variation as produced by DESeq. **Figure S3.** Plot of the base variance as produced by DESeq. **Figure S4.** ECDF plots for the two samples as produced by DESeq. **Table S1.** Unique Sequence Descriptions for Up-Regulated Sequences at P < 0.01. **Table S2.** Unique Sequence Descriptions for Down-Regulated Sequences at P < 0.01. **Table S3.** Site history of hypoxia. **Table S4.** RIN values for RNA samples Click here for file

## References

[B1] Lehr B Bristol S Possolo A Oil Budget Calculator, Deepwater Horizon 2010 50

[B2] Atlas RM Hazen TC Oil biodegradation and bioremediation: a tale of the two worst spills in u.s. History Environ Sci Technol 2011 45 6709 6715 10.1021/es2013227 21699212 PMC3155281

[B3] de Gouw JA Middlebrook AM Warneke C Ahmadov R Atlas EL Bahreini R Blake DR Brock CA Brioude J Fahey DW Organic aerosol formation downwind from the Deepwater Horizon oil spill Science (New York, NY) 2011 331 1295 1299 10.1126/science.1200320 21393539

[B4] Diaz JH The legacy of the Gulf oil spill: analyzing acute public health effects and predicting chronic ones in Louisiana Am J Disaster Med 2011 6 5 22 21466025

[B5] Finch BE Wooten KJ Smith PN Embryotoxicity of weathered crude oil from the Gulf of Mexico in mallard ducks (Anas platyrhynchos) Environ Toxicol Chem 2011 30 1885 1891 10.1002/etc.576 21560150

[B6] Fodrie FJ Heck KL Response of Coastal Fishes to the Gulf of Mexico Oil Disaster PLoS One 2011 6 e21609 10.1371/journal.pone.0021609 21754992 PMC3130780

[B7] Grattan LM Roberts S Mahan WT McLaughlin PK Otwell WS Morris JG The Early Psychological Impacts of the Deepwater Horizon Oil Spill on Florida and Alabama Communities Environ Health Perspect 2011 119 838 843 10.1289/ehp.1002915 21330230 PMC3114820

[B8] Wirgin I Waldman JR Resistance to contaminants in North American fish populations Mutat Res 2004 552 73 100 10.1016/j.mrfmmm.2004.06.005 15288543

[B9] Whitehead A The evolutionary radiation of diverse osmotolerant physiologies in killifish (*Fundulus sp.*) Evolution 2010 64 7 2070 2085 20100216 10.1111/j.1558-5646.2010.00957.x

[B10] Birzele F Schaub J Rust W Clemens C Baum P Kaufmann H Weith A Schulz TW Hildebrandt T Into the unknown: expression profiling without genome sequence information in CHO by next generation sequencing Nucleic Acids Res 2010 38 3999 4010 10.1093/nar/gkq116 20194116 PMC2896516

[B11] Siebert S Robinson MD Tintori SC Goetz F Helm RR Smith SA Shaner N Haddock SHD Dunn CW Differential Gene Expression in the Siphonophore Nanomia bijuga (Cnidaria) Assessed with Multiple Next-Generation Sequencing Workflows PLoS One 2011 6 e22953 10.1371/journal.pone.0022953 21829563 PMC3146525

[B12] Fraser BA Weadick CJ Janowitz I Rodd FH Hughes KA Sequencing and characterization of the guppy (Poecilia reticulata) transcriptome BMC Genomics 2011 12 202 10.1186/1471-2164-12-202 21507250 PMC3113783

[B13] Williams D Brown S Crawford D Contemporary and historical influences on the genetic structure of the estuarine-dependent Gulf killifish Fundulus grandis Mar Ecol Prog Ser 2008 373 111 121

[B14] Karchner SI Powell WH Hahn ME Identification and functional characterization of two highly divergent aryl hydrocarbon receptors (AHR1 and AHR2) in the teleost Fundulus heteroclitus. evidence for a novel subfamily of ligand-binding basic helix loop helix-Per-ARNT-Sim (bHLH-PAS) factors J Biol Chem 1999 274 33814 33824 10.1074/jbc.274.47.33814 10559277

[B15] Oleksiak MF Karchner SI Jenny MJ Franks DG Welch DBM Hahn ME Transcriptomic assessment of resistance to effects of an aryl hydrocarbon receptor (AHR) agonist in embryos of Atlantic killifish (Fundulus heteroclitus) from a marine Superfund site BMC Genomics 2011 12 263 10.1186/1471-2164-12-263 21609454 PMC3213123

[B16] Whitehead A Dubansky B Bodinier C Garcia TI Miles S Pilley C Raghunathan V Roach JL Walker N Walter RB Science Applications in the Deepwater Horizon Oil Spill Special Feature: Genomic and physiological footprint of the Deepwater Horizon oil spill on resident marsh fishes Proc Natl Acad Sci 2011 [ahead of print] 10.1073/pnas.1109545108 PMC3528528 21949382

[B17] Fath DM Kong X Liang D Lin Z Chou A Jiang Y Fang J Caro J Sang N Histone deacetylase inhibitors repress the transactivation potential of hypoxia-inducible factors independently of direct acetylation of HIF-alpha J Biol Chem 2006 281 13612 13619 10.1074/jbc.M600456200 16543236 PMC1564196

[B18] Nizet V Johnson RS Interdependence of hypoxic and innate immune responses Nat Rev Immunol 2009 9 609 617 10.1038/nri2607 19704417 PMC4343208

[B19] White KL An overview of immunotoxicology and carcinogenic polycyclic aromatic hydrocarbons Environmental Carcinogenesis Reviews 1986 4 2 163 202 10.1080/10590508609373342

[B20] van der Meer DLM van den Thillart GEEJM Witte F de Bakker MAG Besser J Richardson MK Spaink HP Leito JTD Bagowski CP Gene expression profiling of the long-term adaptive response to hypoxia in the gills of adult zebrafish Am J Physiol Regul Integr Comp Physiol 2005 289 R1512 R1519 10.1152/ajpregu.00089.2005 15994372

[B21] Hochachka PW Buck LT Doll CJ Land SC Unifying theory of hypoxia tolerance: molecular/metabolic defense and rescue mechanisms for surviving oxygen lack P Natl Acad Sci USA 1996 93 9493 9498 10.1073/pnas.93.18.9493 PMC38456 8790358

[B22] Thomas P Teleost model for studying the effects of chemicals on female reproductive endocrine function The Journal of experimental zoology Supplement : published under auspices of the American Society of Zoologists and the Division of Comparative Physiology and Biochemistry/the Wistar Institute of Anatomy and Biology 1990 4 126 128 1974774 10.1002/jez.1402560421

[B23] Su Z Li Z Chen T Li Q-Z Fang H Ding D Ge W Ning B Hong H Perkins RG Comparing next-generation sequencing and microarray technologies in a toxicological study of the effects of aristolochic acid on rat kidneys Chem Res Toxicol 2011 24 1486 1493 10.1021/tx200103b 21834575

[B24] Oleksiak MF Roach JL Crawford DL Natural variation in cardiac metabolism and gene expression in Fundulus heteroclitus Nat Genet 2005 37 67 72 15568023 10.1038/ng1483 PMC1447534

[B25] Oleksiak MF Churchill GA Crawford DL Variation in gene expression within and among natural populations Nat Genet 2002 32 261 266 10.1038/ng983 12219088

[B26] Rees BB Andacht T Skripnikova E Crawford DL Population proteomics: quantitative variation within and among populations in cardiac protein expression Mol Biol Evol 2011 28 1271 1279 10.1093/molbev/msq314 21109588

[B27] Zerbino DR McEwen GK Margulies EH Birney E Pebble and rock band: heuristic resolution of repeats and scaffolding in the velvet short-read de novo assembler PLoS One 2009 4 e8407 10.1371/journal.pone.0008407 20027311 PMC2793427

[B28] Zerbino DR Birney E Velvet: algorithms for de novo short read assembly using de Bruijn graphs Genome Res 2008 18 821 829 10.1101/gr.074492.107 18349386 PMC2336801

[B29] Langmead B Trapnell C Pop M Salzberg SL Ultrafast and memory-efficient alignment of short DNA sequences to the human genome Genome Biol 2009 10 R25 10.1186/gb-2009-10-3-r25 19261174 PMC2690996

[B30] Anders S Huber W Differential expression analysis for sequence count data Genome Biol 2010 11 R106 10.1186/gb-2010-11-10-r106 20979621 PMC3218662

[B31] Darling AE Carey L Feng W-C The Design, Implementation, and Evaluation of mpiBLAST ClusterWorld Conference & Expo and the 4th International Conference on Linux Clusters 2003 San Jose, California: The HPC Revolution: 2003

[B32] Conesa A Götz S Blast2GO: A Comprehensive Suite for Functional Analysis in Plant Genomics International journal of plant genomics 2008 2008 619832 18483572 10.1155/2008/619832 PMC2375974

[B33] Conesa A Götz S García-Gómez JM Terol J Talón M Robles M Blast2GO: a universal tool for annotation, visualization and analysis in functional genomics research Bioinformatics (Oxford, England) 2005 21 3674 3676 10.1093/bioinformatics/bti610 16081474

[B34] Götz S García-Gómez JM Terol J Williams TD Nagaraj SH Nueda MJ Robles M Talón M Dopazo J Conesa A High-throughput functional annotation and data mining with the Blast2GO suite Nucleic Acids Res 2008 36 3420 3435 10.1093/nar/gkn176 18445632 PMC2425479

