# Supplementary material for: RNA-Seq reveals complex genetic response to deepwater horizon oil release in Fundulus grandis
Source: BMC Genomics. 2012 Sep 12;13:474. doi: 10.1186/1471-2164-13-474 (PMC3487974; doi:10.1186/1471-2164-13-474)
Supplement: Additional file 1 — Figure S1. Length/quantity statistics of assemblies for odd values of K from 21 to 49. In each graph that has two plots, the blue describes the full set of contigs while the red describes only those contigs 500bp or over. Figure S2. Plot of squared coefficient of variation as produced by DESeq. Figure S3. Plot of the base variance as produced by DESeq. Figure S4. ECDF plots for the two samples as produced by DESeq. Table S1. Unique Sequence Descriptions for Up-Regulated Sequences at P < 0.01. Table S2. Unique Sequence Descriptions for Down-Regulated Sequences at P < 0.01. Table S3. Site history of hypoxia. Table S4. RIN values for RNA samples [file 1471-2164-13-474-S1.doc]

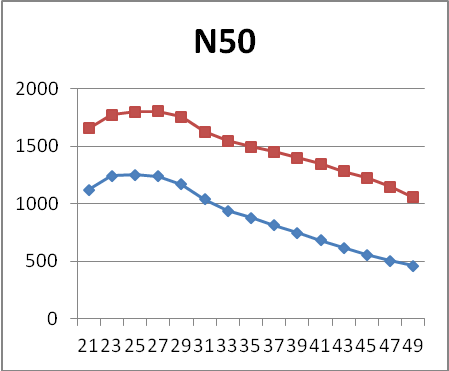

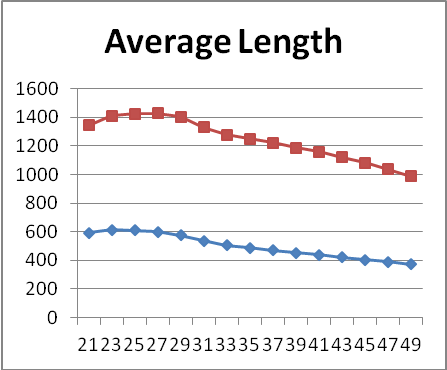

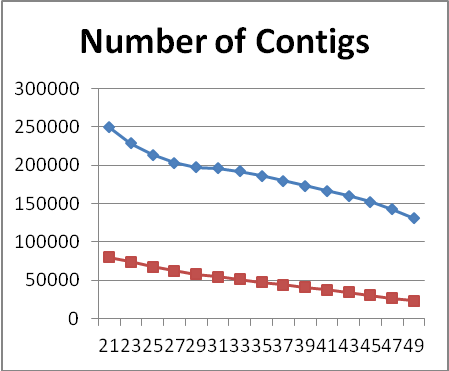

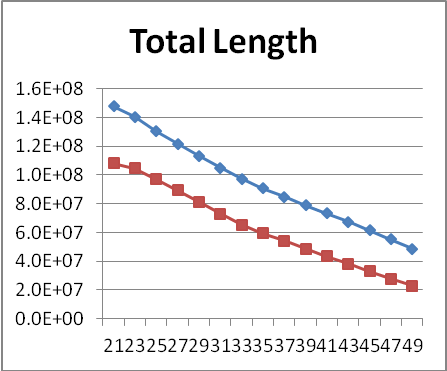

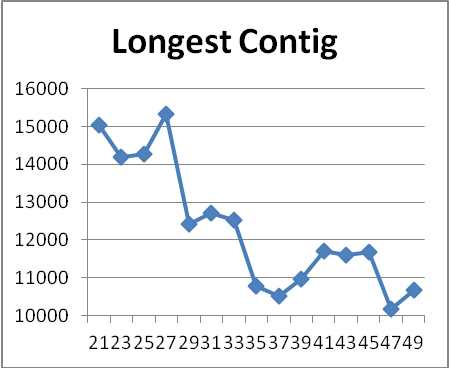


Figure S1. Length/quantity statistics of assemblies for odd values of K from 21 to 49.
In each graph that has two plots, the blue describes the full set of contigs while the red describes only those contigs 500bp or over.

Figure S2. Plot of squared coefficient of variation as produced by DESeq


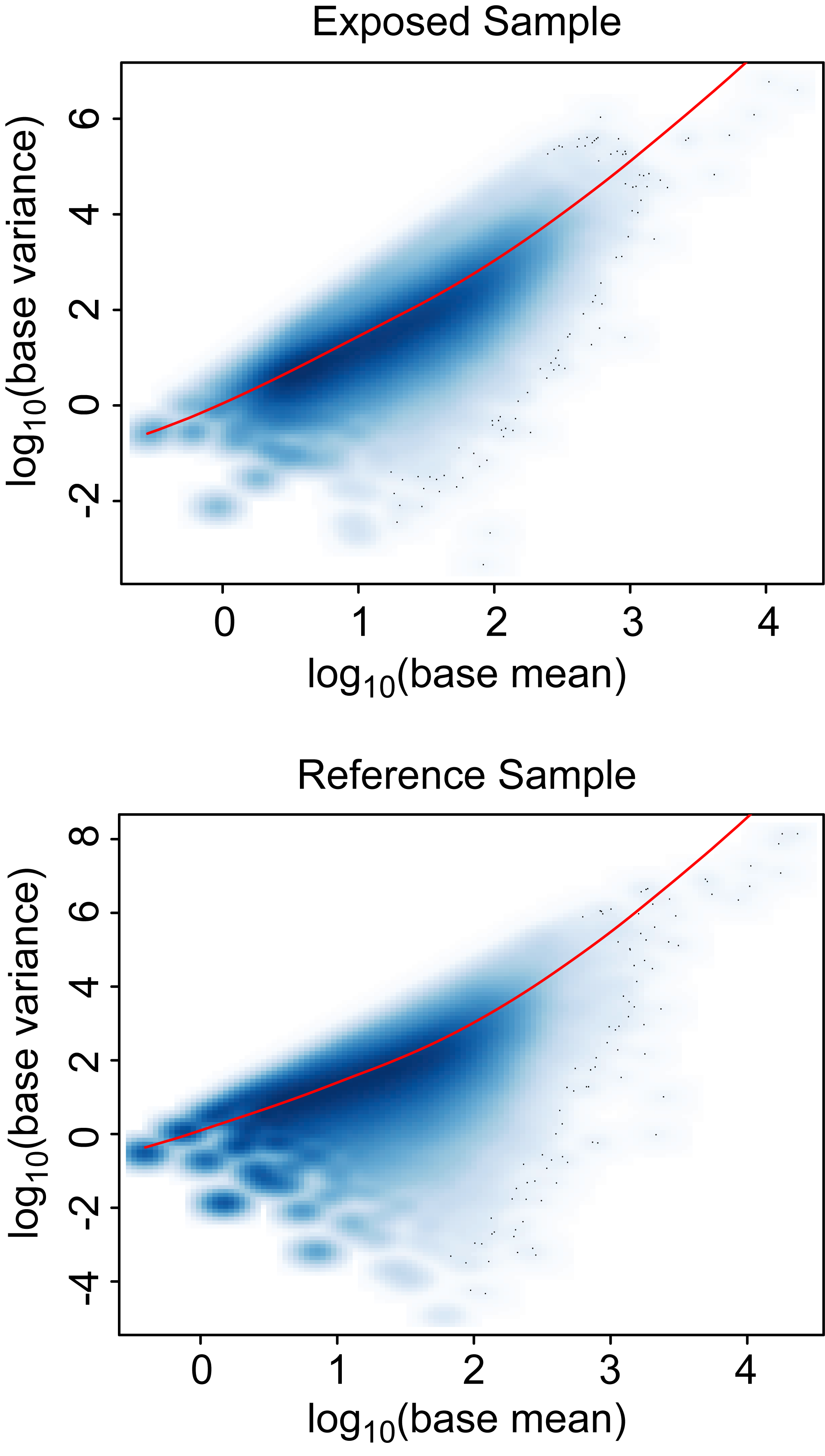


Figure S3. Plot of the base variance as produced by DESeq

Figure S4. ECDF plots for the two samples as produced by DESeq.

Table S1. Unique Sequence Descriptions for Up-Regulated Sequences at P < 0.01

_6- partial

_6-like

14-3-3 protein

1-acylglycerol-3-phosphate o-acyltransferase 9

1-acyl-sn-glycerol-3-phosphate acyltransferase theta-b

24 kda secreted protein

2610204m08rik protein

40s ribosomal protein s27

4-aminobutyrate mitochondrial precursor

4-hydroxybutyrate coenzyme a transferase

5 -3 exoribonuclease 1

5 -3 exoribonuclease partial

52 kda repressor of the inhibitor of the protein kinase (p58ipk-interacting protein) (58 kda interferon-induced protein kinase-interacting protein) (death-associated protein 4) (thap domain-containing protein 0)

60 kda heat shock mitochondrial precursor

60s acidic ribosomal protein p0

60s ribosomal protein l7

6-phosphofructo-2-kinase fructose- -biphosphatase 1

a chain high-resolution structure of ascaris trypsin inhibitor in solution: direct evidence for a ph induced conformational transition in the reactive site

a kinase anchor protein 2

a kinase anchor protein 9 isoform 2

a230067g21rik protein

aaa family protein

abhydrolase domain containing 6

abhydrolase domain-containing protein 6

ac1147-like protein

acetyl- carboxylase 2

acetyl ldl receptor

acetyl- mitochondrial precursor

acetyl-coa synthetase

acetyl-coenzyme a carboxylase alpha

acetyl-coenzyme a carboxylase beta

acetyl-coenzyme a synthetase

acn9 protein mitochondrial precursor

actin

activin a type iia precursor

activin a type iib

activin beta e

activin d precursor

activin d-like

acyl- -binding protein

acyl-coenzyme a binding domain containing 5 isoform 1

acyl-coenzyme a oxidase 1

acyl-coenzyme a oxidase isoform cra_a

acyl-coenzyme a oxidase palmitoyl

adam metallopeptidase domain 9

adam metallopeptidase with thrombospondin type 1 13

adamts-like 1

adducin 3

adenosine kinase

adenosine kinase a

adenosine monophosphate deaminase 2 (isoform l)

adenylate kinase 1

adp atp translocase

adp-ribosylation factor 4

ae binding protein 1

af126830_1c-type lectin tc-ctl-4

af296376_1mhc class i heavy chain

af355375_1 reverse transcriptase

af370723_1intracellular globin

af4 fmr2 member 4

af465280_1coagulation factor v precursor

af523312_1oncosphere-specific antigen

agrin precursor

ahnak nucleoprotein

a-kinase anchor protein 9

a-kinase anchoring protein 220

akt substrate as250

al2sf_human ame: full=amyotrophic lateral sclerosis 2 chromosomal region candidate gene 16 protein

alanine aminotransferase 2

aldhehyde dehydrogenase family subfamily a1

aldolase c

aldolase fructose-bisphosphate

all-1 related protein

alpha 1c

alpha 3 type vi collagen

alpha 8 like 4

alpha accessory factor 132- partial

alpha hemoglobin a

alpha thalassemia mental retardation syndrome x-linked (rad54 cerevisiae)

alpha v (vitronectin alpha antigen cd51)

alpha-2-macroglobulin receptor

alpha-subunit of prolyl 4-hydroxylase

alternative oxidase

aminocarboxymuconate semialdehyde decarboxylase

aminoglycoside phosphotransferase domain-containing protein 1-like

ammecr1 protein

and pleckstrin domain protein 1

angiopoietin-related protein 4

angiopoietin-related protein 4 precursor

angiotensin ii type 2

ani s 9 allergen precursor

ankyrin repeat and ibr domain-containing protein 1

ankyrin repeat-containing cofactor-1

ankyrin-1 isoform 9

antigenic determinant of reca protein homolog

anti-mullerian hormone

apical protein

apolipoprotein b

apoptosis regulator bcl-x

aquaporin 9a

arrestin domain containing 3

arrestin domain-containing protein 3

art3_yeast ame: full=uncharacterized protein art3 ame: full=antisense to ribosomal rna transcript protein 3

aryl hydrocarbon receptor 1b

aryl hydrocarbon receptor 2

ash1 ( or homeotic)-like

aspartyl protease family member (asp-2)

at rich interactive domain 1c

at rich interactive domain 2

at rich interactive domain 4b (rbp1-like)

at rich interactive domain 5b (mrf1-like)

at5g12010 f14f18_180

ataxin 7

at-hook-containing transcription factor 1 (embryonic large molecule derived from yolk sac)

atp synthase f0 subunit 6

atpase family aaa domain-containing protein 2

atpase family aaa domain-containing protein 2b

atpase subunit 6

atpase type 13a3

atp-binding sub-family a member 1

atp-binding sub-family a member 3

atp-binding sub-family a member 4

atp-binding sub-family b (mdr tap) member 11

atp-binding sub-family c (cftr mrp) member 12

atp-binding sub-family c (cftr mrp) member 4

atp-binding sub-family c (cftr mrp) member 9

atp-binding sub-family c (cftr mrp) member isoform cra_a

atp-binding sub-family d member 2

atp-dependent dna helicase homolog ( cerevisiae)

attractin

autosomal dominant 5

axin 1

baculoviral iap repeat-containing 6

band 3 anion exchange protein

bat2 domain containing 1

b-cell cll lymphoma 11a

b-cell lymphoma 6 protein

b-cell lymphoma 6 protein homolog

b-cell lymphoma 6 protein isoform 1

benzodiazapine receptor associated protein 1

beta 5

beta a

beta ab

beta globin

beta-tubulin isotype 1

beta-tubulin isotype 2

bile salt export pump

biliverdin reductase a

bone morphogenetic protein 1

bone morphogenetic protein 1a

brahma protein 1

brain and muscle arnt-like 1 protein

brct domain protein

briggsae cbr-abf-2 protein

briggsae cbr-asp-6 protein

briggsae cbr-ben-1 protein

briggsae cbr-calu-1 protein

briggsae cbr-clec-1 protein

briggsae cbr-col-40 protein

briggsae cbr-cpl-1 protein

briggsae cbr-dhs-11 protein

briggsae cbr-dhs-20 protein

briggsae cbr-enol-1 protein

briggsae cbr-ges-1 protein

briggsae cbr-gst-11 protein

briggsae cbr-mce-1 protein

briggsae cbr-mmcm-1 protein

briggsae cbr-oat-1 protein

briggsae cbr-pes-9 protein

briggsae cbr-sod-1 protein

briggsae cbr-ttr-33 protein

briggsae cbr-ubq-1 protein

briggsae cbr-vit-2 protein

bromodomain and wd repeat domain containing 2

bromodomain phd finger transcription factor

brugia malayi antigen

cadherin 5

calcitonin receptor-like

calcium atpase

calcium voltage- beta 1 subunit

calcium-activated potassium channel beta 2 subunit

calmodulin binding protein 4

calpain 1 catalytic subunit

calpain 7

calpain-1 catalytic subunit

calponin transgelin

calumenin

camp responsive element binding protein 3-like 3

cap-gly domain containing linker protein 1 isoform 1

capicua homolog

capn1 protein

carboxypeptidase d

carboxypeptidase member 1

carboxypeptidase member 1-like

carboxypeptidase-like protein x2

cardiac muscle 1

carnitine palmitoyltransferase 1a

cas-br-m ecotropic retroviral transforming sequence

cathepsin l

cbp p300-interacting transactivator with glu asp-rich carboxy-terminal domain 1

ccctc-binding factor

cd200 molecule

cd302 antigen precursor

cdc37 cell division cycle 37 homolog ( cerevisiae)

cdk5 regulatory subunit-associated protein 2

cell adhesion molecule 2a

cell wall-associated hydrolase

centromere protein 350 400ka

centrosomal protein 192kda

centrosomal protein 2

cerebral endothelial cell adhesion molecule 1

cgmp-stimulated cyclic nucleotide phosphodiesterase

chaperonin cpn60

chemokine (c-c motif) receptor-like 1

chloride channel 6

chromodomain helicase dna binding protein 7

chromodomain helicase dna-binding protein 3 short isoform

chromosome 1 open reading frame 9

chromosome 12 open reading frame 28

chromosome 12 open reading frame 51

chromosome 20 open reading frame 3

chromosome 9 open reading frame 125

circadian clock protein per2

class i helical cytokine receptor number 29

class member 1

classical mhc class i antigen

clock protein

clock-3a protein

clusterin

c-myc promoter binding protein isoform 1

c-myc promoter-binding protein irlb

cndp dipeptidase 2 (metallopeptidase m20 family)

coagulation factor v

coiled-coil and c2 domain containing 1b

cold shock domain containing rna binding

collagen alpha-6 chain precursor

collagen prolyl 4-hydroxylase alpha iii subunit

collagen type vi alpha 4

collagen type vi alpha 4-like

complement c1q tumor necrosis factor-related protein 5 precursor

complement receptor-like

connexin 43

conserved hypothetical protein [Talaromyces stipitatus ATCC 10500]

contactin associated 5

copia-type -like

cordon-bleu homolog

coxsackie virus and adenovirus receptor

cre-

creb binding protein

cryptochrome 1

cryptochrome 2a

c-type lectin

cuticle collagen

cuticular collagen

c-x-c motif chemokine 14 precursor

cxorf33 precursor

cxxc finger 6

cyclin d1

cyclin i

cyclin-dependent kinase-like 1

cyclophilin-type peptidyl-prolyl cis-trans isomerase- bmcyp-2

cystathionine gamma-lyase

cysteine- angiogenic 61

cytochrome b

cytochrome c oxidase subunit i

cytochrome c oxidase subunit ii

cytochrome c oxidase subunit iii

cytochrome family subfamily polypeptide 1

cytochrome family subfamily polypeptide 2

cytochrome family subfamily polypeptide 23

cytochrome family subfamily polypeptide 24

cytochrome family subfamily polypeptide 27

cytochrome family subfamily polypeptide 29

cytochrome oxidase subunit 1

cytochrome oxidase subunit ii

cytochrome p450

cytochrome p450 2p3

cytochrome p450 cyp2n

cytochrome p450 family 1 subfamily b polypeptide 1

cytochrome p450 like_tbp

cytoplasmic actin

cytoplasmic dynein 1 heavy chain 1

cytoplasmic intermediate filament protein

cytoplasmic linker associated protein 2

cytoskeletal actin 2

cytosolic malate dehydrogenase

cytosolic purine 5-nucleotidase

d5ertd579e protein

dead (asp-glu-ala-asp) box polypeptide 3

death inducer-obliterator 1

dedicator of cytokinesis 5

dedicator of cytokinesis 7

dedicator of cytokinesis 8

dedicator of cytokinesis 9

defensin beta 2

delta 3

denn madd domain containing 4a

denn madd domain containing 4c

deoxyribonuclease ii beta

dep domain containing 6

der_and-312 phosphate carrier

diaphanous homolog 2

dicer protein

dihydrolipoamide dehydrogenase

dihydrolipoyl mitochondrial precursor

dihydropyridine receptor beta 1c subunit

dip2 disco-interacting protein 2 homolog b

disabled homolog 2

disabled homolog 2 interacting protein

dishevelled associated activator of morphogenesis 2

disintegrin and metalloproteinase domain-containing protein 10

disks large-associated protein 1 isoform 1

dispatched homolog 1

dmx-like 1

dmx-like 1 isoform 1

dmx-like isoform cra_a

dmx-like isoform cra_c

dna helicase hel308

dna polymerase zeta catalytic subunit

dna strand-exchange protein sep1 isoform 2

dna-damage-inducible transcript 4

dna-dependent protein kinase catalytic subunit

dopamine receptor d1

dopey family member 1

dopey family member 2

dot1- histone h3 methyltransferase ( cerevisiae)

down-regulated in metastasis

dynamin 2

dysferlin (dystrophy associated fer-1-like protein) (fer-1 like protein 1)

dysferlin isoform 6

dysferlin isoform cra_a

dystonin

dystroglycan 1

e1a binding protein isoform cra_b

e1a binding protein p300

e1a binding protein p400

e2f-associated phosphoprotein

ecto-nox disulfide-thiol exchanger 1

ectonucleoside triphosphate diphosphohydrolase 1

ectonucleotide pyrophosphatase phosphodiesterase 5 ( function)

egl nine homolog 3

egl nine homolog 3 ( elegans)

eh-domain containing 3

elastin microfibril interfacer 1a

elastin microfibril interfacer 1b

elegans protein confirmed by transcript evidence

elegans protein partially confirmed by transcript evidence

elongation factor 1 alpha

elongation factor 2

elongation factor 2 kinase (eef-2 kinase) (eef-2k) (calcium calmodulin-dependent eukaryotic elongation factor 2 kinase)

elongation factor rna polymerase ii

elongation factor tu gtp binding domain containing 1

elongation factor-2 kinase

endonuclease reverse transcriptase

endothelial cell adhesion molecule

enolase family member (enol-1)

enzymatic - partial

epithelial v-like antigen 1

epsin 1

epsin 1 isoform 1

ero1-like ( cerevisiae)

erythrocyte membrane protein band

erythrocyte membrane protein band -like 2

ester hydrolase c11orf54 homolog

estrogen receptor beta 2

ethylmalonic encephalopathy 1

ets variant gene 4 (e1a enhancer binding e1af)

ets2 repressor factor

eukaroytic elongation factor 2 kinase

eukaryotic translation elongation factor 2

eukaryotic translation initiation factor 5a

excretory secretory c-type lectin tes-32

exocyst complex component 7

f5 protein

fad-dependent oxidoreductase domain containing 2

family with sequence similarity member a

family with sequence similarity member a1

family with sequence similarity member b

fas binding factor 1

fat tumor suppressor homolog 1

fatty acid 2-hydroxylase

fatty acid desaturase domain member 6

fatty acid elongation protein 3

fatty acid retinoid binding protein

fc receptor-like 2-like

feline sarcoma oncogene

fibronectin 1

fibronectin 1b

fibronectin leucine rich transmembrane protein 2

fibronectin type iii and spry domain containing 1-like

fibronectin type iii domain containing 3b

filamin beta isoform 3

fk506 binding protein 12-rapamycin associated protein 1

fk506 binding protein 5

flavin containing monooxygenase 5

flavin-containing monooxygenase 13

flavin-containing monooxygenase fmo1

flavoprotein subunit of complex ii

fn1 protein

fragile site-associated protein

fras1 related extracellular matrix 1

fructose-bisphosphate aldolase

fructose-bisphosphate aldolase c

fumarate hydratase

fumarylacetoacetate hydrolase domain-containing protein 1

furry homolog

furry homolog-like

fyve and coiled-coil domain containing 1

g protein beta 1 subunit

g protein-coupled receptor 1

g protein-coupled receptor 126

g protein-coupled receptor 64

gag-pol poly

galactocerebrosidase precursor

gamma 3

gamma s3

ganab protein

gap-pol poly

glb_psedc ame: full=extracellular globin flags: precursor

glis family zinc finger 2

glis2- partial

globoside alpha- -n-acetylgalactosaminyltransferase 1

glucagon-like peptide 2 receptor

glutamate dehydrogenase

glutamate mitochondrial

glutamate receptor interacting protein 1

glutathione peroxidase

glutathione transferase

glyceraldehyde-3-phosphate dehydrogenase

glycerophosphodiester phosphodiesterase domain containing 1

glycogen synthase kinase 3 alpha

glycosyltransferase 25 domain containing 1

glyoxalase domain containing 1

glyoxylase 1

golgi golgin subfamily macrogolgin (with transmembrane signal) 1

golgi golgin subfamily macrogolgin (with transmembrane signal) isoform cra_a

growth factor receptor-bound protein 10

growth hormone receptor

growth regulation by estrogen in breast cancer-like

gtpase activating rap domain-like 1

guanine nucleotide binding protein (g protein) alpha 12

guanine nucleotide exchange factor p532 isoform 1

guaninenucleotide-binding protein beta subunit

hat family dimerisation domain containing partial

hbba_serqu ame: full=hemoglobin subunit beta-a ame: full=hemoglobin beta-a chain ame: full=beta-a-globin

heat repeat containing 5b

heat repeat containing 7a

heat shock protein 30

heat shock protein 60 kda

heat shock protein 70

heat shock protein 90

heat shock protein beta-1

heat shock protein family member (hsp-25)

heavy chain non-muscle

heavy polypeptide a

heavy polypeptide non-muscle

heavy polypeptide non-muscle isoform 7

hect (homologous to the e6-ap carboxyl terminus) domain and rcc1 -like domain 1

hect domain and rcc1-like domain 1

hect domain and rld 2

hect domain containing 1

helicase with zinc finger

hemoglobin alpha chain

hemoglobin beta chain

hemoglobin beta-a chain

hemoglobin subunit alpha

hemoglobin subunit beta

heparin-binding growth factor 1

hepatic leukemia factor- partial

histone deacetylase 4

histone h3 methyltransferase dot1 variant a

histone h5a

histone -like protein

hla-b associated transcript 2

hmg-box transcription factor 1

homeobox containing 1

homeodomain interacting protein kinase 2

hook homolog 3

hormone-sensitive lipase

host cell factor 1

host cell factor c1

hspd1 protein

huntingtin

huntingtons disease protein

hyaluronidase-2 precursor

hydroxyacyl glutathione hydrolase

hydroxyacylglutathione hydrolase

hypermethylated in cancer 1

hypermethylated in cancer 2

hypothetical kda protein in chromosome

hypothetical protein [Brugia malayi]

hypothetical protein [Pectinaria gouldii]

hypothetical protein Bm1_05555 [Brugia malayi]

hypothetical protein Bm1_06125 [Brugia malayi]

hypothetical protein Bm1_35525 [Brugia malayi]

hypothetical protein BRAFLDRAFT_124497 [Branchiostoma floridae]

hypothetical protein BRAFLDRAFT_199990 [Branchiostoma floridae]

hypothetical protein C17F4.7 [Caenorhabditis elegans]

Hypothetical protein CBG18225 [Caenorhabditis briggsae]

hypothetical protein G11MC16DRAFT_3625 [Geobacillus sp. G11MC16]

hypothetical protein LOC572528 [Danio rerio]

hypothetical protein LOC768199 [Danio rerio]

hypothetical protein PABG_06807 [Paracoccidioides brasiliensis Pb03]

hypothetical protein SORBIDRAFT_0016s002240 [Sorghum bicolor]

hypothetical protein SORBIDRAFT_0070s002020 [Sorghum bicolor]

hypothetical protein Ssol98_08391 [Sulfolobus solfataricus 98/2]

hypothetical protein TcasGA2_TC004229 [Tribolium castaneum]

hypothetical protein TcasGA2_TC005078 [Tribolium castaneum]

hypothetical protein Y67D2.3 [Caenorhabditis elegans]

hypothetical upf0041 protein in chromosome

hypoxia-inducible factor 2 alpha

imap family member 4- partial

immunoglobulin heavy chain variable region

immunoglobulin i-set domain containing protein

immunoglobulin light chain precursor

immunoglobulin m heavy chain secreted form

immunoglobulin mu heavy chain

immunoglobulin mu heavy chain secreted form

immunoglobulin superfamily member 4b

in family member (ttn-1)

independent phosphoglycerate mutase isoform 1

inorganic p hosphatase family member (pyp-1)

inositol -triphosphate type 2

inositol -triphosphate type partial

insulin receptor b

insulin-like growth factor 2 receptor

integrin beta 3b

inter-alpha trypsin inhibitor heavy chain precursor 5

interleukin 11 alpha

intermediate filament protein

intersectin 1 (sh3 domain protein)

inverted formin-2

iq motif and sec7 domain 2

isoform 1

isoform 2

isoform b

isoform cra_a

itchy e3 ubiquitin protein ligase homolog

jnk-interacting leucine zipper protein long form

jumonji domain containing 3

k+-dependent na+ ca+ exchanger related-protein

kaiso-like zinc finger protein

kda protein in nof-fb transposable element

keratin 8

kh domain containing protein

kiaa0100 protein

kiaa0232 gene product

kiaa0399 protein

kiaa0785 protein

kiaa0809 protein

kiaa1840 protein

kinase insert domain receptor like

kinesin family member 13b

kinesin family member 1b

krueppel-like factor 6

kruppel-like factor 10

kruppel-like factor 13

kruppel-like factor 4

kynureninase (l-kynurenine hydrolase)

l _3-like

lactate dehydrogenase-a

laminin gamma 1

large subunit ribosomal protein 10

large tumor homolog 2

latent transforming growth factor beta binding protein 1

latent transforming growth factor beta binding protein 2- partial

latent transforming growth factor beta binding protein 4

lats homolog 1-like

layilin a

lemur tyrosine kinase 2

lepre1 protein

leprecan 1

leucine rich protein

leucine rich repeat containing 16a

leucine rich repeat containing 8 member a

leucine-rich repeat kinase 2

leucine-rich repeat transmembrane protein flrt2 precursor (fibronectin-like domain-containing leucine-rich transmembrane protein 2)

leucine-rich repeat-containing protein 68

leukemia inhibitory factor receptor

leukemia inhibitory factor receptor alpha

leukemia-associated protein with a cxxc domain

leukocyte receptor cluster member 8 homolog

lfa-3(delta tm)

lfi-1

ligand-dependent partial

light chain kinase

like 2

limb girdle muscular dystrophy 2b (autosomal recessive) partial

lin-5 interacting protein family member (lfi-1)

lipid phosphate phosphatase-related protein type 2

lipoma preferred partner lpp

lipoprotein lipase

lipoprotein receptor-related protein

lman2l protein

loc398083 protein

loc398474 protein

loc493204 protein

loc495463 protein

loc553527 protein

loc558045 protein

loc559330 protein

loc559360 protein

loc733209 protein

loc779081 protein

loc779595 protein

loc794108 protein

loc798746 protein

low density lipoprotein receptor adaptor protein 1

low density lipoprotein receptor-related protein 1

low density lipoprotein receptor-related protein 5

lps-responsive vesicle beach and anchor containing isoform 2

lrrfip1a protein

lysine-specific demethylase 5c

lysophosphatidic acid receptor 2

lysosomal membrane glycoprotein 2

lysyl hydroxylase

lysyl hydroxylase 1

lysyl oxidase

lysyl oxidase-like 2

maf protein

major histocompatibility class i receptor

malate cytoplasmic

male germ cell-associated kinase

male-specific protein

mannose c type 1

mannose c type 1-like 1

map microtubule affinity-regulating kinase 1

mapk8 protein

matrix metallopeptidase 15 (membrane-inserted)

mediator complex subunit 13

member ras oncogene family

membrane associated guanylate ww and pdz domain containing 1

membrane associated guanylate ww and pdz domain containing 3

membrane-spanning 4-domains subfamily a member 8a-like

meningioma (disrupted in balanced translocation) 1

meningioma expressed antigen 5 isoform 1

met proto-oncogene precursor

methyl- binding domain protein 5

methylmalonic aciduria (cobalamin deficiency) cbld with homocystinuria

methylmalonyl coenzyme a mutase

methylmalonyl epimerase

mgc53127 protein

mgc53303 protein

mgc53694 protein

mhc class i antigen

mhc class ii antigen beta chain

mib2 protein

microfilament and actin filament cross-linker protein

microtubule-actin crosslinking factor 1

microtubule-actin crosslinking factor 1-like

microtubule-associated protein 4

microtubule-associated protein tau-like

midasin (midas-containing protein) isoform 2

midasin homolog

misshapen-like kinase 1 isoform cra_b

mitochondrial 28s ribosomal protein s33

mitochondrial carrier protein flj44862 homolog

mitochondrial trans-2-enoyl- isoform cra_b

mitogen-activated protein kinase 12

mitogen-activated protein kinase kinase kinase 13

mitogen-activated protein kinase kinase kinase kinase 3

mitogen-activated protein kinase kinase kinase kinase 4

mkiaa0230 protein

mkiaa0429 protein

mkiaa0633 protein

mkiaa1042 protein

monocarboxylate transporter 10

monoglyceride lipase

morc family cw-type zinc finger 3

mpv17 kidney disease mutant-like -like

mtap4 protein

multiple egf-like-domains 8

multiple pdz domain protein

mutated in colorectal cancers

myelin protein zero-like 1

myeloid ecotropic viral integration site 1 homolog 2 isoform cra_b

myeloid lymphoid or mixed-lineage leukemia (trithorax drosophila) translocated 4

myeloid lymphoid or mixed-lineage leukemia 2

myeloid lymphoid or mixed-lineage leukemia 3

myeloid lymphoid or mixed-lineage leukemia 3-like

myeloid lymphoid or mixed-lineage leukemia 4a

myh9 protein

myo-2

myocyte enhancer factor 2d

myomesin member 3

myosin 18a

myosin 18a isoform b isoform 1

myosin heavy chain

myosin id

myosin light polypeptide 1

myosin phosphatase-rho interacting protein isoform 2

myosin regulatory light chain

myosin tail family protein

myosin xviiia

myosin-ixb- partial

mysp_anisi ame: full=paramyosin ame: allergen=ani s 2

myst histone acetyltransferase (monocytic leukemia) 3

myst histone acetyltransferase (monocytic leukemia) 4

myst4_macfa ame: full=histone acetyltransferase myst4 short=myst-4 ame: full= ybf2 sas2 and tip60 protein 4

nacht- lrr- and pyd-containing protein 1 paralog b

nadh dehydrogenase 1 alpha subcomplex 4

nadh dehydrogenase subunit 4

necap endocytosis associated 1

nematode cuticle collagen n-terminal domain containing protein

n-ethylmaleimide-sensitive factor attachment beta

netrin 1

netrin 1a

neural adhesion molecule

neural cell adhesion molecule 1

neuraminidase 1

neurobeachin-like 1

neuroblastoma-amplified protein

neurofibromatosis 1

neurofibromin 1 ( von recklinghausen watson disease) isoform cra_a

neurogenic locus notch homolog protein 3 precursor

neuron navigator 2 isoform 2

neutral alpha-glucosidase ab-like

nfix protein

nicotinamide riboside kinase 2

nipped-b homolog isoform cra_a

nlr pyrin domain containing 1-like

nlr pyrin domain containing 6-like

nmda receptor-regulated gene 1a

non-erythrocytic 1

nonmuscle myosin heavy chain

normal mucosa of esophagus-specific gene 1 protein

notch homolog 2

notch homolog translocation-associated

notch-3 homolog

novel alpha-globin

novel gene

novel nacht domain containing

novel protein

novel protein containing a latrophilin cl-1-like gps domain

novel protein containing a lectin c-type domain

novel protein cytochrome family subfamily j

novel protein dna polymerases

novel protein early endosome antigen 1

novel protein glutaminase

novel protein homeodomain interacting protein kinase

novel protein human and mouse leishmanolysin-like (metallopeptidase m8 family)

novel protein human and mouse sema transmembrane domain and cytoplasmic 6d

novel protein human ras association ( af-6) and pleckstrin homology domains 1

novel protein mitogen-activated protein kinase kinase 7

novel protein mouse silencing mediator of retinoic acid and thyroid hormone receptor

novel protein podocalyxin-like

novel protein ranbp2-like and grip domain containing

novel protein rho rac guanine nucleotide exchange factor 18

novel protein spectrin repeat nuclear envelope 1

novel protein vertebrate angiopoietin-like 4 ( im:7144703)

novel protein vertebrate apolipoprotein b (including ag antigen)

novel protein vertebrate carnitine palmitoyltransferase 1a

novel protein vertebrate chondroitin sulfate proteoglycan 4 (melanoma-associated)

novel protein vertebrate creb binding protein (rubinstein-taybi syndrome)

novel protein vertebrate cytoplasmic linker associated protein 1

novel protein vertebrate dab2 interacting protein

novel protein vertebrate hla-b associated transcript 2

novel protein vertebrate iq motif and sec7 domain 1

novel protein vertebrate kinesin family member 1 family

novel protein vertebrate lps-responsive vesicle beach and anchor containing

novel protein vertebrate microtubule-actin crosslinking factor 1

novel protein vertebrate misshapen-like kinase 1

novel protein vertebrate myeloid lymphoid or mixed-lineage leukemia (trithorax drosophila)

novel protein vertebrate myeloid lymphoid or mixed-lineage leukemia (trithorax drosophila) translocated 1 ( zgc:136639)

novel protein vertebrate phosphoinositide-3- class alpha polypeptide

novel protein vertebrate plectin intermediate filament binding protein 500kda

novel protein vertebrate rev1 homolog ( cerevisiae)

novel protein vertebrate ring finger protein 144

novel protein vertebrate sh3 domain and tetratricopeptide repeats 1

novel protein vertebrate solute carrier family 26

novel protein vertebrate spectrin repeat nuclear envelope 1

novel protein vertebrate spectrin repeat nuclear envelope 2

novel protein vertebrate syntaxin binding protein 5

novel protein with atpase family associated with various cellular activities domains

novel protein zebrafish hemoglobin alpha-adult 1

novel rna helicase family protein

novel ubiquitin-protein ligase

nuclear factor 1 x-type

nuclear factor erythroid 2-related factor 1

nuclear factor i isoform cra_c

nuclear receptor binding set domain protein 1 isoform 1

nuclear receptor coactivator 2

nuclear receptor coactivator 6

nuclear receptor co-repressor 2

nucleic acid binding protein

nucleosome assembly protein

one cut family member 2

open reading frame 34

orf2-encoded protein

organic anion transporting polypeptide 1b3

orphan g-protein coupled receptor dez

ovary-specific c1q-like factor

oxidation resistance 1

oxoglutarate (alpha-ketoglutarate) dehydrogenase

oxoglutarate dehydrogenase

p300 protein

p53-associated parkin-like cytoplasmic protein isoform 1

paired box gene 6a

palmitoyl- oxidase

palmitoyl-protein thioesterase 2

pantothenate kinase 1

par-3 partitioning defective 3 homolog ( elegans)

patatin-like phospholipase domain containing 7

paternally expressed 10 isoform 1

pc4 and sfrs1 interacting protein 1

pdz domain-containing guanine nucleotide exchange factor pdz-gef2

pebph_caeel ame: full=phosphatidylethanolamine-binding protein homolog

pecanex-like 3 isoform 2

pepsin inhibitor

peptidase family m28 containing protein

peptidyl arginine type ii

peptidylarginine deiminase

peptidylprolyl isomerase b

pericentrin b

peroxidasin homolog

peroxisomal trans-2-enoyl- reductase

peroxisome proliferative activated coactivator beta

phophatidylinositol 3-kinase

phosphatidic acid phosphatase type 2

phosphatidylethanolamine-binding protein

phosphatidylinositol 3- alpha polypeptide

phosphatidylinositol 3-kinase regulatory subunit beta

phosphatidylinositol 4- alpha polypeptide isoform 2

phosphatidylinositol 4-kinase iii alpha-like

phosphatidylinositol 4-kinase type 3 alpha

phosphatidylinositol glycan anchor class m

phosphatidylinositol n-acetylglucosaminyltransferase subunit p

phosphatidylinositol-4-phosphate 5- type gamma

phosphatidylinositol-binding clathrin assembly

phosphodiesterase 4a-like

phosphodiesterase 4d interacting protein

phosphodiesterase camp-specific (phosphodiesterase e4 dunce homolog)

phosphodiesterase cgmp-stimulated

phosphoenolpyruvate carboxykinase

phosphoglycerate kinase

phosphoglycerate mutase 1

phosphoinositide-3- alpha polypeptide

phosphoinositide-3- class 2 alpha polypeptide

phospholipase epsilon 1

phosphoprotein enriched in astrocytes 15

pi-3-kinase-related kinase smg-1

pigment epithelium-derived factor

plasma glutamate carboxypeptidase

platelet derived growth factor receptor alpha

platelet-activating factor isoform beta subunit 30kda

pleckstrin homology domain family member 3

pleckstrin homology domain interacting protein

plectin-1 isoform 1hij

plexin b1

plexin b2

plexin b3

pogo transposable element with krab domain

pol polyprotein

pol protein

poliovirus receptor-related 1a

poliovirus receptor-related 3

poliovirus receptor-related 3-like

pol-like protein

poly (adp-ribose) polymerase member 3

poly (adp-ribose) polymerase member 4

polycomb group protein asxl2

polycystin 1

polymeric immunoglobulin receptor

polyprotein [Atlantic salmon swim bladder sarcoma virus]

polyubiquitin precursor

polyunsaturated fatty acid elongase

potassium channel interacting protein 1

potassium large conductance calcium-activated subfamily alpha member 1

potassium large conductance calcium-activated subfamily beta member 2

potassium voltage-gated kqt-like member 1

potassium voltage-gated subfamily member 7 isoform 2

potential phospholipid-transporting atpase id (atpase class i type 8b member 2)

ppar-alpha interacting complex protein 285

pr gag-pro-pol

predicted protein [Micromonas pusilla CCMP1545]

predicted protein [Trichoplax adhaerens]

PREDICTED: hypothetical protein [Danio rerio]

PREDICTED: hypothetical protein [Saccoglossus kowalevskii]

PREDICTED: hypothetical protein [Strongylocentrotus purpuratus]

PREDICTED: hypothetical protein LOC325536 [Danio rerio]

PREDICTED: ReO_6-like [Danio rerio]

PREDICTED: similar to predicted protein [Hydra magnipapillata]

p-rex2 protein

prion-like-(q n-rich)-domain-bearing protein family member (pqn-22)

probable e3 ubiquitin-protein ligase mycbp2 (myc-binding protein 2) (protein associated with myc) (pam highwire rpm-1 protein)

procollagen- 2-oxoglutarate 4-dioxygenase (proline 4-hydroxylase) alpha polypeptide i

procollagen- 2-oxoglutarate 5-dioxygenase 3

procollagen- -oxoglutarate 5-dioxygenase 1 precursor

proline rich 12

proline serine-rich coiled-coil 2

prolyl 4- alpha ii subunit isoform 1 precursor

prolyl 4-hydroxylase subunit alpha-2

prolyl 4-hydroxylase subunit alpha-2 precursor

promethin variant 2

propionyl coenzyme a beta polypeptide

propionyl-coenzyme a alpha polypeptide

protein

protein 7 transactivated by hepatitis b virus x antigen

protein cgmp- type i

protein cxorf17 homolog isoform cra_a

protein daple (dvl-associating protein with a high frequency of leucine residues) (coiled-coil domain-containing protein 88c) (hook-related protein 2)

protein disulfide isomerase

protein disulphide isomerase

protein dpy-19 homolog 3

protein kinase alpha

protein kinase c substrate 80k-h

protein phosphatase 1f (pp2c domain containing)

protein phosphatase regulatory subunit 10

protein phosphatase regulatory subunit 14c

protein tyrosine non-receptor type 23

protein tyrosine receptor b

protein tyrosine receptor j

protein wiz (widely-interspaced zinc finger-containing protein) (zinc finger protein 803)

protein-tyrosine kinase

protocadherin 2a3-like

protoporphyrinogen oxidase

pr-set7-like

psd-95 sap90-associated protein-2

ptk7 protein tyrosine kinase 7 precursor

putative transposase [Pleuronectes platessa]

pyridoxal kinase

r3h domain containing 2

r3h domain-containing protein 2

rap guanine nucleotide exchange factor 2

rap1gds1 protein

ras association and pleckstrin homology domains 1 isoform 2

ras protein activator like 2- partial

ras responsive element binding protein 1

ras-like family 11 member a

rcc2 homolog

rearranged l-myc fusion

reelin isoform 2

reelin isoform a isoform 3

reelin precursor

regulator of g-protein signaling 9

-related lipid transfer protein 5

ReO_6 [Oryzias latipes]

replicase helicase endonuclease-like

reticulocalbin ef-hand calcium binding domain

retinoic acid induced 17

retinol dehydrogenase 13

retrotransposon nucleocapsid protein

retrotransposon-like family member (retr-1)-like

retrovirus -like

rev3- catalytic subunit of dna polymerase zeta

reverse transcriptase

reverse transcriptase ribonuclease h methyltransferase-like

reverse transcriptase-like protein

reversion-inducing-cysteine-rich protein with kazal motifs

rho gtpase activating protein 4

rho gtpase activating protein 5

rho guanine nucleotide exchange factor 11

rho guanine nucleotide exchange factor 17

rho-type gtpase-activating protein flj32810-like isoform 2

ribosomal protein l15

ribosomal protein s2

riken cdna 1300002k09 gene

riken cdna 2310021p13 gene

riken cdna a930016p21

ring finger and ccch-type zinc finger domains 1

ring finger protein 145

ring finger protein 24

rna binding motif and elmo ced-12 domain 1

rna binding single stranded interacting protein

rna binding single stranded interacting protein 2

round spermatid basic protein 1-like

rrna promoter binding protein

rsph3 protein

s1 rna binding domain 1

sam domain and hd domain 1

sam domain and hd domain-containing protein 1

sam domain and hd domain-containing protein 1 (dendritic cell-derived ifng-induced protein) (monocyte protein 5) (mop-5)

sam domain and hd partial

saps domain member 2

scavenger receptor class member isoform cra_b

selenoprotein m precursor

sema immunoglobulin domain transmembrane domain and short cytoplasmic 4c

sema immunoglobulin domain transmembrane domain and short cytoplasmic 4g

semaphorin receptor

senescence-associated protein

septin 5

serine dehydratase

serine incorporator 5

serine peptidase 1

serine protease

serine protease kunitz type 1 b

serine threonine kinase 38 like

serine threonine-protein kinase pim-1

serine threonine-protein kinase sik2

serotonin transporter

set binding factor 1

set binding protein 1

set domain containing 1a

set domain containing 5

set domain-containing protein 5

sfrs1 protein

sh3-domain binding protein 4

sh3-domain grb2-like 1

si:ch211- isoform 1

si:dkey- isoform 2

si:dkey- protein

sideroflexin 1

signal cub egf-like 1

signal transducer and activator of transcription 5

sister of p-glycoprotein

sj965_schja ame: full=upf0506 protein sjchgc02965 flags: precursor

sjchgc01393 protein

slc13a2-prov protein

slc43a2 protein

slit homolog 2

slit2 protein

slit2-a protein

smarcc1 protein

smoothelin-like 2

snap25-interacting protein

snf2-related cbp activator protein

snrnp70 protein

sodium hydrogen exchanger isoform 3

sodium-dependent phosphate transporter 2

soluble calcium-activated nucleotidase 1

solute carrier family 13 (sodium-dependent dicarboxylate transporter) member 2

solute carrier family 16 (monocarboxylic acid transporters) member 1

solute carrier family 16 (monocarboxylic acid transporters) member 12

solute carrier family 22 member 6

solute carrier family 23 (nucleobase transporters) member 2

solute carrier family 25 (mitochondrial carrier phosphate carrier) member 23

solute carrier family 25 (mitochondrial carrier phosphate carrier) member 25

solute carrier family 27 (fatty acid transporter) member 2

solute carrier family 27 member 2

solute carrier family 5 (sodium glucose cotransporter) member 2

solute carrier family 5 (sodium-dependent vitamin transporter) member isoform cra_a

solute carrier family 6 (neurotransmitter creatine) member 8

solute carrier family 6 (neurotransmitter gaba) member 13

solute carrier family 9 (sodium hydrogen exchanger) member 6

solute carrier family member 1

solute carrier family member 10 (aromatic amino acid transporter)

solute carrier family member 12 (monocarboxylic acid transporter 12)

solute carrier family member 1a

solute carrier family member 1b

solute carrier family member 23-like

solute carrier family member 3

solute carrier family member 36

solute carrier family member 4

solute carrier family member 4-like

solute carrier family member 5

solute carrier family member 5-like

solute carrier organic anion transporter family member 2a1-like

solute carrier organic anion transporter member 1c1

solute carrier organic anion transporter member 1c1 isoform 1

somatostatin receptor type 2

son of sevenless homolog 2

sorting nexin 9-like

soul protein

spastic ataxia of charlevoix-saguenay

spectrin alpha 2

spectrin beta 2

spen transcriptional regulator

sphingosine-1-phosphate phosphatase 1

spry domain containing 3

stabilin 2

strawberry notch homolog 2

strawberry notch homolog isoform cra_c

subfamily member 13

subfamily member 4

subfamily member a2

subfamily member isoform cra_a

succinate dehydrogenase iron-sulfur mitochondrial precursor

succinic semialdehyde dehydrogenase

sulfate transporter

swi snf matrix actin dependent regulator of subfamily member 2

sxp ral-2 family protein 2 isoform 1

synaptojanin 1

synaptotagmin-like 2-like

talin 2

tao kinase 1

tar1_klula ame: full=protein tar1

tbt-binding protein 2

tc1-like transporase

temporarily assigned gene name family member (tag-163)-like

tensin 1

tensin 3

testis expressed sequence 264

testis flippase

tet oncogene family member 2-like

tet oncogene family member 3

tetratricopeptide repeat domain partial

tgf-beta receptor type iii precursor (tgfr-3) (transforming growth factor beta receptor iii)

thap domain-containing protein 9

thiamine triphosphatase-like

thioredoxin family protein

thrombospondin 1

thyroid hormone receptor alpha

thyroid hormone receptor alpha b

thyroid hormone receptor beta

thyroid hormone receptor interactor 11

tight junction protein 1 (zona occludens 1) protein

tight junction protein 1-like

tpa: tpa_exp: replicase helicase endonuclease

tpa: tpa_inf: eukaryotic translation elongation factor 1a

tpa_exp: transposase

tpm_anisi ame: full=tropomyosin ame: allergen=ani s 3

transcription factor

transcription factor 7-like 1-a

transcription factor mtf-1

transferrin receptor 2

transformation transcription domain-associated protein

transformation transcription domain-associated protein isoform 1

transforming growth beta receptor i

transforming sequence-like

transient receptor potential cation subfamily member 1

transketolase

transketolase isoform 1

translocated promoter region (to activated met oncogene)

translocator protein

transmembrane activator and caml interactor

transmembrane protein 14c

transmembrane protein 16c

transmembrane protein 189

transmembrane protein 4

transmembrane protein 41a

transmembrane protein 63b

transmembrane protein c1orf78 homolog

transmembrane protein loc124446

transposable element tc1 transposase

transposable element tcb1 transposase

transposase

transposase [Pleuronectes platessa]

transposase [Salmo salar]

transposase domain-containing protein

transposase homolog

transposon tx1 uncharacterized 149 kda

transthyretin-like family protein

trinucleotide repeat containing 6a

trinucleotide repeat containing 6a isoform 2 isoform 5

tripartite motif protein trim29-like

tripartite motif protein trim4-like

tripartite motif-containing 71

triple functional domain (ptprf interacting)

tropomodulin 1

troponin c

troponin family protein

troponin t

truncated poly

tubby like protein 4

tumor necrosis factor receptor member 14 (herpesvirus entry mediator)

tumor necrosis factor receptor member 21

type alpha 1

type alpha 3

type ii cytoskeletal 8

type v p-type atpase

tyrosine hydroxylase 2

uba and wwe domain containing 1

ubiquitin family protein

ubiquitin protein ligase e3 component n-recognin 4

ubiquitin protein ligase e3 component n-recognin 5

ubiquitin protein ligase e3b

ubiquitin specific peptidase 32

ubiquitin specific peptidase 34

ubiquitin specific peptidase x- isoform 1

ubiquitin specific protease 24

ubiquitin specific protease 34

ubiquitin-like with phd and ring finger domains 2

udp glucuronosyltransferase 1 family polypeptide a1

udp glucuronosyltransferase 5 family polypeptide g1

udp glycosyltransferase 1 polypeptide a1-like

udp-glucuronosyl transferase

udp-glucuronosyltransferase

udp-glucuronosyltransferase 1

udp-glucuronosyltransferase 2a1-like

ugt1ab protein

ugt3_plepl ame: full=udp-glucuronosyltransferase short=udpgt

unc-13 homolog b ( elegans)

unc-51-like kinase 1

unc-93 homolog b1 ( elegans)

uncharacterized protein c1orf71

uncoordinated family member (unc-87)

unknown [Glycine max]

unknown [Xiphophorus maculatus]

unknown [Zea mays]

unnamed protein product [Tetraodon nigroviridis]

upf0041 domain protein

urocanase domain containing 1

utrophin

vacuolar protein sorting 13 homolog a

vacuolar protein sorting 13 homolog a ( cerevisiae)

vacuolar protein sorting 13 homolog d ( cerevisiae)

vacuolar protein sorting 13b isoform 2 isoform 4

vacuolar protein sorting 13c

vacuolar protein sorting 13c protein

vacuolar protein sorting 13d

vasa

very large inducible gtpase-1-like

very long-chain acyl- synthetase

viral a-type inclusion protein

vitamin d receptor a

vitellogenin structural genes (yolk protein genes) family member (vit-6)

voltage-dependent calcium channel gamma-5 subunit

voltage-gated sodium channel

von willebrand factor

von willebrand factor type egf and pentraxin domain containing 1

vps10 domain receptor protein sorcs 3-like

v-src sarcoma (schmidt-ruppin a-2) viral oncogene homolog

wd repeat and fyve domain containing isoform cra_a

wdr9 form a

widely-interspaced zinc finger motifs

wnk lysine deficient protein kinase 1

wnk lysine deficient protein kinase isoform cra_d

wnk1 protein

wu: partial

ww and c2 domain containing 1

xenotropic and polytropic retrovirus receptor

xk-related protein 8

zbed1 protein

zbtb44 protein

zfhx2 protein

zgc:158345 protein

zgc:161969 protein

zgc:163027 protein

zgc:171579 protein

zgc:174680 protein

zgc:174908 protein

zinc bed-type containing 4

zinc cchc domain containing 24

zinc dhhc-type containing 23

zinc finger and btb domain containing 16

zinc finger and btb domain containing 4

zinc finger and btb domain containing 44

zinc finger homeobox 2

zinc finger homeodomain 4

zinc finger partial

zinc finger protein 292

zinc finger protein 318

zinc finger protein 347-like

zinc finger protein 37 homolog

zinc finger protein 395

zinc finger protein 618

zinc finger protein 638-like

zinc finger protein 646

zinc finger protein 704

zinc finger protein isoform cra_b

zinc finger protein isoform cra_c

zinc finger protein mym-type

zinc finger swim domain-containing protein kiaa0913

zinc fyve domain containing 26

zinc miz-type containing 1

zinc mym domain containing partial

zinc zz type with ef hand domain 1

zinc zz-type with ef hand domain 1 isoform 2

zinc-finger homeodomain protein 4-like

zona pellucida glycoprotein 2 prepro

Table S2. Unique Sequence Descriptions for Down-Regulated Sequences at P < 0.01

_6-like

14-3-3 protein

14-alpha demethylase

2 (trypsin 2) isoform 2

2 -cyclic nucleotide 3 phosphodiesterase

24-dehydrocholesterol reductase

2-aminoethanethiol dioxygenase

2-cys peroxiredoxin

2-oxoglutarate and iron-dependent oxygenase domain containing 1

39s ribosomal protein mitochondrial precursor

3-hydroxy-3-methylglutaryl-coenzyme a reductase

3-hydroxy-3-methylglutaryl-coenzyme a reductase a

3-hydroxy-3-methylglutaryl-coenzyme a synthase 1

3-oxo-5-alpha-steroid 4-dehydrogenase 2

40s ribosomal protein s14

40s ribosomal protein s19

40s ribosomal protein s5

40s ribosomal protein s7

4-methyl-5(b-hydroxyethyl)-thiazol monophosphate biosynthesis enzyme

5-hydroxytryptamine receptor 3a

5-methyltetrahydrofolate-homocysteine methyltransferase reductase

5-oxoprolinase (atp-hydrolysing)

60 kda lysophospholipase

60s ribosomal export protein nmd3

60s ribosomal protein l10a

60s ribosomal protein l13a

60s ribosomal protein l14

60s ribosomal protein l26

60s ribosomal protein l27a

60s ribosomal protein l37a

78 kda glucose-regulated protein

a chain crystal structure of an activated (thr-

ac1147-like protein

acetoacetyl- synthetase

acetyl- acetyltransferase 2

acetyl-coenzyme a acyltransferase 1

achain rnase zf-1a

actin binding 1a

actin depolymerizing factor

acyl- desaturase

acyl- synthetase bubblegum family member 2

acyl- synthetase long-chain family member 1

acyl- synthetase long-chain family member 4

acyl- synthetase short-chain family member 2

acyl- synthetase short-chain family member 3

adenine phosphoribosyltransferase

adenosylmethionine decarboxylase 1

adenylosuccinate synthase

adenylylsulfate kinase

adipose triglyceride lipase

adp-ribosyl cyclase 1

adp-ribosylation factor-like 5b

adp-ribosylation factor-like 5c

ae binding protein 2

aebp2 protein

af252633_1alpha amylase

af266241_1tyrosine aminotransferase

af410782_1cardiac and skeletal muscle-specific bop2

ag2_rat ame: full=protein ag2

alanine dehydrogenase

alanine-glyoxylate aminotransferase

alanyl aminopeptidase

alcohol dehydrogenase 8a

alcohol dehydrogenase class-3

aldehyde dehydrogenase

aldehyde dehydrogenase 1b1 precursor isoform 3

aldo-keto reductase

aldose reductase

alkylation repair homolog 3 ( coli)

allograft inflammatory factor 1

alpha 8 like 2

alpha m (complement component 3 receptor 3 subunit)

alpha smooth aorta

aminopeptidase

amme syndrome candidate gene 1

angiopoietin-like 3

angiopoietin-like 7

angiotensin converting enzyme

angiotensin i converting enzyme 2

ankyrin repeat domain 29

ankyrin repeat domain-containing protein 56

anterior gradient protein 2 homolog precursor

antigen processing proteasome-associated protein

apolipoprotein b

apolipoprotein d

apoptosis-inducing factor -like mitchondrion-associated inducer of death isoform 2

apoptosis-inducing factor -like mitochondrion-associated inducer of isoform cra_a

aspa protein

asparagine synthetase

aspartic protease

asteroid homolog 1

atp binding

atp citrate isoform cra_b

atp citrate lyase

atpase aaa domain containing 4

atpase family aaa domain containing 4

atpase family aaa domain-containing protein 2

atpase subunit 6

aurora kinase b

autophagy related 16 like 2-like

autosomal recessive 1a

bactericidal permeability-increasing protein lipopolysaccharide-binding protein

baculoviral iap repeat-containing 5

basic helix-loop-helix member a15

basic transcription factor 3

basic transcription factor 3-like 4

bax inhibitor-1

b-cell translocation gene 3

beta-2 microglobulin

beta-2-microglobulin precursor

beta-actin

beta-carotene -monooxygenase 1

beta-galactoside-binding lectin

bhlh protein dec1a

biotinidase precursor

bloodthirsty [Chaenocephalus aceratus]

bone morphogenetic protein 10

brain specific kinase 146

btb poz domain-containing protein

bystin-like

c14orf159 protein

c1orf51 protein

c1q-like protein

C7orf57 [Anoplopoma fimbria]

c8orf4 homolog

ca++ slow twitch 2

cadherin li cadherin (liver-intestine)

calcium calmodulin-dependent protein kinase id

calcium regulated heat stable protein isoform cra_a

calmodulin-like 4

calpain small subunit 1

camp responsive element modulator

camp-responsive element modulator

carbohydrate (chondroitin 4) sulfotransferase 11

carboxypeptidase polypeptide 2

carotene- -monooxygenase

caspase 8

caspase recruitment domain member 6-like

caspase xa

catechol-o-methyltransferase domain-containing protein 1

cathepsin h

cathepsin h precursor

cation transport regulator-like protein 1

cc chemokine

cc chemokine ck-

c-c motif chemokine 14 precursor

c-c motif chemokine 20 precursor

c-c motif chemokine 25 precursor

ccaat enhancer-binding protein beta 2

ccaat enhancer-binding protein delta

ccr4 carbon catabolite repression 4-like

ccr4 carbon catabolite repression 4-like ( cerevisiae)

cd151 antigen

cd209 antigen-like protein a

cd3 gamma delta

cd38 antigen

cd9 antigen

cd97 antigen-like

cdc-like kinase 1

cdc-like kinase 2

cdc-like kinase 4

cdc-like kinase 4b

cell division cycle associated 7-like

cell division cycle associated 8

centromere protein p

c-fos protein

cg057 protein

cg17150- isoform a

chadl protein

ched related family member (ptr-2)-like

chemokine (c-c motif) receptor 7

chemokine cc-like protein

chemokine cxc-like protein

chemokine receptor 4

chitinase 1

chitotriosidase

cholesterol 7-alpha hydroxylase

cholesterol 7-alpha-monooxygenase

choline-phosphate cytidylyltransferase b isoform 2

cholinergic delta

cholinergic receptor nicotinic alpha polypeptide 1

chromatin assembly factor subunit b isoform cra_a

chromobox homolog 7

chromosome 14 open reading frame 68

chromosome 20 open reading frame 55

cl029_danre ame: full=uncharacterized protein c12orf29 homolog

class iii alcohol chi subunit

class major histocompatibility transactivator

classical mhc class i antigen

claudin 10 like

c-myc-binding protein

coenzyme q3 methyltransferase

coiled-coil domain containing 109b

coiled-coil domain containing 45

coiled-coil domain containing 80

coiled-coil domain containing 86

coiled-coil domain containing 94

coiled-coil domain-containing protein 5

complement component c4

complement factor bf c2

condensin-2 complex subunit h2

conserved hypothetical protein [Toxoplasma gondii GT1]

conserved plasmodium protein

copia-type -like

cornifelin homolog b

creb protein

crel2_danre ame: full=cysteine-rich with egf-like domain protein 2 flags: precursor

ctp synthase 1

c-type lysozyme

cullin-associated nedd8-dissociated protein 1

c-x-c chemokine receptor type variant 1

cyclin a2

cyclin b1

cyclin-dependent kinase inhibitor 1a

cysteine-rich secretory protein 3

cysteine-rich secretory protein 3 isoform 1

cysteine-rich venom protein presursor

cytidine monophospho-n-acetylneuraminic acid synthetase

cytochrome b

cytochrome c oxidase subunit 1

cytochrome c oxidase subunit ii

cytochrome c oxidase subunit iii

cytochrome family subfamily polypeptide 1

cytochrome family subfamily polypeptide 23

cytochrome oxidase subunit 1

cytochrome oxidase subunit 2

cytochrome p450

cytochrome p450 cyp2n

cytochrome p450 cyp2x12

cytokine receptor family member b1

cytokine-dependent hematopoietic cell linker

cytokine-inducible sh2-containing protein

cytoplasmic 1

cytoplasmic thioredoxin isoenzyme of the thioredoxin system

d-amino acid oxidase

death effector domain containing

death effector domain containing 2

death-associated 1-a

dehydrodolichyl diphosphate synthase

dehydrogenase reductase sdr family member 12

delta-6 fatty acyl desaturase

delta-9-desaturase 1

delta-9-desaturase 2

deltex 3-like

deltex homolog 3

denn madd domain containing 2d

deoxycytidylate deaminase

deoxyuridine triphosphatase

desmin

dhe3_chaac ame: full=glutamate mitochondrial short=gdh

diacylglycerol kinase zeta

dihydrofolate reductase

dihydroorotate dehydrogenase

diphosphomevalonate decarboxylase

dna chr brigham & women s genetics 0951 expressed

dna repair protein rad51 homolog a

dna replication complex gins protein psf2

dna replication licensing factor mcm2

dna replication licensing factor mcm5

dna topoisomerase 2-alpha

dna-binding protein inhibitor id-1

dna-damage-inducible transcript 4

dna-damage-inducible transcript 4-like

dna-directed rna polymerase i a

dnase domain containing 3

domain containing 2

dr1-associated corepressor

dual specificity phosphatase 1

dual specificity phosphatase 16

dual specificity phosphatase 19

dual specificity phosphatase 26

dynein light chain axonemal

dyskeratosis congenita dyskerin

dystrophin dp260-2 isoform

e3 ubiquitin-protein ligase lincr

early growth response 1

elongation factor 1-

elongation factor 1-alpha

endonuclease domain-containing 1 protein precursor

endonuclease reverse transcriptase

endoplasmic reticulum aminopeptidase 2

envelope polyprotein

ependymin precursor

ependymin related protein 1

epidermal growth factor receptor

erbb receptor feedback inhibitor 1

estrogen receptor a

estrogen receptor alpha

ethanolamine kinase 1

eukaryotic translation elongation factor 1 beta 2

eukaryotic translation initiation factor 3 subunit e-interacting protein

eukaryotic translation initiation factor 6

extracellular protein with signal 5xegf and apple domains

f115_danre ame: full=protein fam115 ame: full=protein fam139a

family with sequence similarity member a

family with sequence similarity member b

family with sequence similarity member c

farnesyl diphosphate synthase (farnesyl pyrophosphate geranyltranstransferase)

farnesyl pyrophosphate synthetase

farnesyl-diphosphate farnesyltransferase 1

fatty acid delta-6 desaturase

fatty acid synthase

fatty acid-binding intestinal

fatty acyl reductase 1

fatty acyl- reductase 1

f-box and leucine-rich repeat protein 2

f-box and leucine-rich repeat protein 4

f-box and wd-40 domain protein 4

f-box only protein 25

fbp32 precursor

fbp32ii precursor

fc fragment of binding protein

fc receptor-like 2-like

fdps protein

female expressed transcript 1

fibronectin leucine rich transmembrane 1b

fibronectin type iii domain containing 1

fish virus induced trim protein

fk506 binding protein 25kda

flavin containing monooxygenase 5

flavin reductase

fms-related tyrosine kinase 3

folate receptor 1 isoform cra_a

fos-like antigen 2

fra1 protein

frizzled homolog 9

f-type lectin

fumarylacetoacetate hydrolase

g protein-coupled receptor -like

g2 m-phase specific e3 ubiquitin ligase

gag-pol poly

gamma 1 (formerly lamb2) isoform cra_a

gamma-aminobutyric acid rho 1

gap-pol poly

gdp dissociation inhibitor 1

gdp dissociation inhibitor 2

glia maturation factor gamma

glial cell differentiation regulator-like

globoside alpha- -n-acetylgalactosaminyltransferase 1

glucokinase

glucosamine-phosphate n-acetyltransferase 1

glutamate delta 2 interacting protein 1

glutaredoxin-related protein 5

glutathione peroxidase 3

glutathione peroxidase 6

glutathione s-transferase

glutathione s-transferase a

glutathione s-transferase domain-containing protein

glutathione s-transferase m1

glutathione s-transferase theta-1

glutathione transferase omega-1

glutathione-s-transferase theta

glyceraldehyde-3-phosphate dehydrogenase

glycerol kinase

glycerophosphodiester phosphodiesterase domain containing 2-like

glycerophosphodiester phosphodiesterase domain containing 5

glycine amidinotransferase (l-arginine:glycine amidinotransferase)

glycine cleavage system h mitochondrial precursor

glycogenin 1

glypican 5

golgi-localized protein

golgi-localized syntaphilin-related protein

gpi transamidase subunit pig-

granzyme a precursor

grd2i_danre ame: full=delphilin ame: full=glutamate delta 2-interacting protein 1

growth arrest and dna-damage-inducible protein gadd45 alpha

growth arrest and dna-damage-inducible protein gadd45 beta

growth arrest and dna-damage-inducible protein gadd45 gamma

growth differentiation factor 15

gsk-3-binding protein

gtp binding protein 4

gtp cyclohydrolase 1 feedback regulatory protein

gtpase imap family member 7

gtp-binding protein sar1b

guanine nucleotide binding 2

guanylate cyclase beta 3

guanylin precursor

h aca ribonucleoprotein complex subunit 4

h-2 class ii histocompatibility antigen gamma chain

h3 histone family 3a

harbinger transposase derived 1

heart-type fatty acid-binding protein

heat repeat containing 3

heat shock cognate 70

heat shock protein 47

heat shock protein 70

heat shock protein 70 isoform 2

heat shock protein 8

heat shock protein 90

heat shock protein 90 alpha

heat shock protein 90-alpha 2

heat shock protein beta-1

heat shock protein hsp 90-alpha

hect domain and rld 5

hect domain and rld 6

hect type ubiquitin

heg homolog 1

helicase mov-10

heme oxygenase

heme oxygenase 2

hemicentin 1

hepatic leukemia factor-like

herv-h ltr-associating 2

high mobility group

high mobility group protein

high-mobility group 20b

high-mobility group box 2

histidine ammonia-lyase

hmg- reductase

hmgcs1 protein

hsp70-binding protein 1

hspb (heat shock 27kda) associated protein 1

hydin protein

hydroxymethylglutaryl- cytoplasmic

hydroxymethylglutaryl- synthase 1

hydroxymethylglutaryl- synthase 1-like

hydroxysteroid (17-beta) dehydrogenase 7

hypothetical protein [Ictalurus punctatus]

hypothetical protein [Plasmodium berghei strain ANKA]

hypothetical protein [Toxoplasma gondii RH]

hypothetical protein MGL_3949 [Malassezia globosa CBS 7966]

hypothetical protein TGME49_033360 [Toxoplasma gondii ME49]

hypothetical protein TGME49_054910 [Toxoplasma gondii ME49]

hypothetical protein TGME49_073510 [Toxoplasma gondii ME49]

hypothetical protein TGME49_080430 [Toxoplasma gondii ME49]

hypothetical protein TGME49_116630 [Toxoplasma gondii ME49]

hypothetical protein, conserved [Toxoplasma gondii ME49]

ibr domain containing 2

ifit2 protein

imap family member 4-like

immunoglobulin heavy chain

immunoglobulin tau heavy chain membrane-bound form

immunoglobulin tau heavy chain secretory form

immunoresponsive 1 homolog

immunoresponsive gene 1

indoleamine -dioxygenase 1

ing defective family member (mlt-10)- partial

inositol hexaphosphate kinase 2

inositol -trisphosphate 3-kinase c

insect-derived growth factor-a-like protein

insulin precursor

insulin-like growth factor binding protein 1

insulin-like growth factor binding protein 1a

insulin-like growth factor binding protein 2

interferon inducible mx protein

interferon regulatory factor 1

interferon regulatory factor 10

interferon regulatory factor 3

interferon-induced 17 kda protein precursor

interferon-induced 35 kda protein homolog

interferon-induced mx protein

interferon-induced protein 44

interferon-induced protein with tetratricopeptide repeats 5 (ifit-5) (retinoic acid- and interferon-inducible 58 kda protein)

interferon-inducible protein 56

interferon-inducible protein gig1

interferon-inducible protein gig1-like

interferon-inducible protein gig2-like

interferon-inducible protein ifi58-like

interleukin 10 alpha

interleukin 17 receptor b

interleukin 6 signal transducer ( oncostatin m receptor)

interleukin-1 receptor type ii

interleukin-8

interleukin-8-like protein

intraflagellar transport 88 homolog

iodothyronine deiodinase type ii

iodothyronine deiodinase type iii

iron-sulfur cluster assembly enzyme mitochondrial precursor

iron-sulfur cluster scaffold protein

iron-sulfur cluster scaffold variant 2

isoform cra_a

isoform cra_e

isopentenyl-diphosphate delta-isomerase 1

jun b proto-oncogene

k123 protein

karyopherin alpha 2 (rag cohort importin alpha 1)

kda protein

kelch-like 24

keratin 15

kiaa0837 protein

kiaa1277 protein

kiaa1609 protein

kinesin family member 11

kinesin family member c1

kinetochore protein nuf2

krt4 protein

kruppel-like factor 9

l _3-like

lag-3 protein

lamc1 protein

lamina-associated polypeptide 2 isoform beta

laminin b2

laminin gamma 1

laminin subunit gamma-1 precursor

l-amino acid oxidase

lanosterol synthase

lantibiotic synthetase component c-like 1

latrophilin receptor family member (lat-1)-like

lens intrinsic membrane protein

lethal giant larvae homolog 2

leucine rich repeat containing 2

leucine rich repeat containing 39

leukocyte elastase inhibitor

leukocyte immune-type receptor

lin-52 homolog

line-1 reverse transcriptase homolog

lipin 1

lipopolysaccharide-binding protein bactericidal permeability-increasing protein

lipopolysaccharide-induced tumor necrosis factor-alpha factor homolog

liver-expressed antimicrobial peptide 2

loc100126649 protein

loc100137715 protein

loc402847 protein

loc495281 protein

loc558601 protein

loc733436 protein

loc795096 protein

loc799646 protein

long-chain-fatty-acid-- ligase 4

longevity-assurance domain-containing protein

low density lipoprotein receptor adaptor protein 1

l-threonine dehydrogenase

ly6 plaur domain-containing protein 2

lymphocyte-specific protein tyrosine kinase

lysosomal alpha-glucosidase precursor

mad2 mitotic arrest deficient-like 1

major facilitator superfamily domain-containing protein

malic enzyme nadp(+)- mitochondrial

maltase- intestinal

maltase- partial

maltase-glucoamylase (alpha-glucosidase) isoform cra_a

mammalian ependymin-related protein 1 precursor

mannose-1-phosphate guanyltransferase alpha-a

map kinase interacting serine threonine kinase 1

matrix metalloproteinase 9

meiosis inhibitor 1

melanoma differentiation associated protein-5

member ras oncogene family

membrane alanine aminopeptidase

membrane frizzled-related protein

membrane palmitoylated

membrane palmitoylated 1

membrane-spanning 4-domains subfamily a member 8a-like

membrane-toll-like receptor 5

meprin beta

methionine adenosyltransferase alpha

methionine aminopeptidase 1

methyltransferase like 1

mevalonate decarboxylase

mevalonate kinase

mgc81587 protein

mgc82534 protein

mgc83562 protein

mgc83638 protein

mgc89155 protein

mhc class i alpha antigen

mhc class i antigen

mhc class ii antigen-associated invariant chain

mical-like protein 1 (molecule interacting with rab13) isoform 2

microfibril-associated glycoprotein 4 precursor

microtubule aggregate protein homolog

microtubule associated serine threonine kinase-like

mid1-interacting protein 1

minichromosome maintenance complex component 3

minichromosome maintenance complex component 4

minichromosome maintenance complex component 7

mitogen-activated protein kinase 13

mitogen-activated protein kinase 6

mitogen-activated protein kinase kinase kinase 8

mki67 fha domain-interacting nucleolar phospho

mlf1 interacting

mlx interacting protein

mothers against decapentaplegic homolog 7

motile sperm domain containing 1

mterf domain containing 3

muc2 protein

mucolipin 2

multiple egf-like-domains 10

myelin gene regulatory factor

myelin-associated glycoprotein

myelin-oligodendrocyte glycoprotein precursor

myeloid cell lineage chitinase

myoglobin

myosin light polypeptide 6

myotubularin-related protein 1

na k 2cl cotransporter

na k cl cotransporter

n-acylneuraminate cytidylyltransferase

nad kinase

nadh dehydrogenase subunit 1

nadh dehydrogenase subunit 2

nadh dehydrogenase subunit 3

nadh dehydrogenase subunit 4

nadh dehydrogenase subunit 4l

nadh dehydrogenase subunit 5

natriuretic peptide receptor type-c

natt4_thani ame: full=natterin-4 flags: precursor

nattectin precursor

ncl protein

neuraminidase 2

neuraminidase 2-like

neuraminidase 3-like

neuraminidase isoform cra_a

nfx1-type zinc finger-containing protein 1

ngfi-a binding protein 1 (egr1 binding protein 1)

nicotinamide nucleotide transhydrogenase

nicotinamide riboside kinase 2

nima-related kinase 4-like

nipa-like domain containing 2

nitric oxide synthase 1 adaptor protein

nk-lysin type 1

nlr card domain containing 5

nlr pyrin domain containing 1-like

nlr pyrin domain containing 6-like

nol1 nop2 sun domain member 5

non-neuronal tryptophan hydroxylase 1

non-protein coding rna 153

non-smc condensin i subunit d2

non-smc condensin ii subunit h2

nop16 nucleolar protein homolog

notochord-related protein

novel gene

novel immune-type receptor

novel immune-type receptor 1

novel immune-type receptor 25

novel immune-type receptor 8

novel immune-type receptor 9

novel krab box and zinc c2h2 type domain containing protein

novel member of the trypsin family of serine proteases-like isoform 1

novel nacht domain containing

novel protein

novel protein (zgc:162944)

novel protein (zgc:63972)

novel protein arylamine n-acetyltransferases

novel protein bloodthirsty

novel protein glutaminase

novel protein human and mouse nima (never in mitosis gene a)-related kinase 1

novel protein human angiopoietin-like angptl

novel protein non-vertebrate chitin synthase protein

novel protein nucleolin ( zgc:152810)

novel protein vertebrate bai1-associated protein 2-like 2

novel protein vertebrate cdc-like kinase 2

novel protein vertebrate dishevelled associated activator of morphogenesis 1

novel protein vertebrate heat shock 70kda protein 12a

novel protein vertebrate hect domain and rld 3

novel protein vertebrate ngfi-a binding protein 1 (egr1 binding protein 1)

novel protein vertebrate titin

novel serine protease protein

novel ubiquitin-protein ligase

n-terminal ef-hand calcium-binding protein 1-like

nuclear factor interleukin-3-regulated protein

nuclear factor related to kappab binding protein

nuclear protein 1

nuclear receptor binding protein

nuclear receptor subfamily group member 1

nuclear receptor subfamily group member 2

nuclear receptor subfamily group member isoform cra_a

nuclear transport factor 2

nuclear vcp-like

nucleic acid binding protein

nucleobindin 2b

nucleolar protein 5a

nucleoplasmin-like protein no29

nucleoredoxin

nucleoside diphosphate kinase

nucleoside phosphorylase

nusap1 protein

oocyst wall

opioid growth factor receptor

organic-cation transporter-like 3 (solute carrier family 22 member 13)

orm1-like 3 ( cerevisiae)

ornithine carbamoyltransferase

ornithine decarboxylase

orphan nuclear receptor dax-1

ortholog of human family with sequence similarity member a fam20a

otu ubiquitin aldehyde binding 2

ovary-specific c1q-like factor

oxidative stress induced growth inhibitor 1

p97 protein

pan domain protein

pancreatic protein with two somatomedin b domains

patched domain containing 3

patched family protein

pcna-associated factor

pdgfa associated protein 1

pdja1 chaperone

peptide transporter

peptide yy

perforin-1 precursor

period 4

peroxisome proliferator-activated receptor coactivator-related 1

peter pan homolog

phenazine biosynthesis-like domain-containing protein 2

phosphate carrier mitochondrial precursor

phosphatidic acid phosphatase type 2b

phosphoenolpyruvate carboxykinase

phosphoenolpyruvate cytosolic

phosphoethanolamine n-

phosphoethanolamine n-methyltransferase 3

phosphofurin acidic cluster sorting protein 2

phospholipase a2

phospholipase c-like 2- partial

phospholipase d member 4

phosphoribosyl pyrophosphate amidotransferase

phosphoribosyl pyrophosphate synthetase-associated protein 1

phosphoribosylaminoimidazole phosphoribosylaminoimidazole succinocarboxamide synthetase

phosphotriesterase-related protein

phosvitinless vitellogenin

piggybac transposable element derived 1-like

pim-3 oncogene

plakophilin 1

pleckstrin sec7 and coiled-coil binding protein

pml-rara-regulated adapter molecule 1

pol-like protein

polo-like kinase 1

poly (adp-ribose) polymerase member 12

poly (adp-ribose) polymerase member 12b

poly (adp-ribose) polymerase member 14

poly (adp-ribose) polymerase member 14-like

polymeric immunoglobulin receptor

polyprotein

polyserase-2 precursor

popeye 1

predicted protein [Trichoplax adhaerens]

PREDICTED: hypothetical protein [Danio rerio]

PREDICTED: predicted protein-like [Danio rerio]

PREDICTED: similar to transposase [Strongylocentrotus purpuratus]

PREDICTED: wu:fb54e10 [Danio rerio]

PREDICTED: zonadhesin-like [Danio rerio]

pregnancy specific beta-1-glycoprotein 1-like

preprosomatostatin 1a

preprosomatostatin ii

pristanoyl acyl-coenzyme a oxidase 3

probable atp-dependent rna helicase dhx58

probable dimethyladenosine transferase

probable e3 ubiquitin-protein ligase rnf144a-a

profilin

programmed cell death protein 6

progranulin type i

prolyl endopeptidase

properdin p factor complement 2 precursor

proteasome activator complex subunit 1

proteasome beta type 10

proteasome beta type 8

proteasome subunit alpha type-6

proteasome subunit beta type 10

proteasome subunit beta type-7 precursor

protein arginine methyltransferase 3

protein ef-hand calcium binding domain 2

protein phosphatase 1h (pp2c domain containing)

protein trs85 homolog

protein zyg-11 partial

protocadherin lkc

proto-oncogene protein c-fos

prune homolog

ptc7 protein phosphatase homolog ( cerevisiae)

pumilio homolog 2

purine nucleoside phosphorylase

purinergic receptor ligand-gated ion 5

purinergic receptor p2x3

pyrroline-5-carboxylate reductase 1

pyrroline-5-carboxylate reductase family member 2 variant 1

pyruvate liver and rbc

quinolinate phosphoribosyltransferase-like

radical s-adenosyl methionine domain containing 2

radical s-adenosyl methionine domain-containing protein 2

rcc1 domain containing 1

receptor-transporting protein 3

refractile-body associated protein

regulator of chromosome condensation and btb domain containing protein 1

regulator of g-protein signalling 3

rep78 protein

replication protein 70kda

reticulon 1

retinoic acid receptor responder protein 3

retinol binding protein cellular

retinol dehydrogenase 12

retinol dehydrogenase 12 (all-trans and 9-cis)

retrotransposon-like family member (retr-1)-like

reverse

reverse transcriptase

reverse transcriptase-like protein

rho family gtpase 1

rho gtpase-activating protein 15

ribonuclease inhibitor-like

ribonuclease subunit b

ribonucleotide reductase m2 polypeptide

ribosomal protein

ribosomal protein l10a

ribosomal protein l13a

ribosomal protein l15

ribosomal protein l17

ribosomal protein l18

ribosomal protein l22-like 1

ribosomal protein l23a

ribosomal protein l4

ribosomal protein l6

ribosomal protein l7

ribosomal protein l7a

ribosomal protein l8

ribosomal protein l9

ribosomal protein s11

ribosomal protein s16

ribosomal protein s20

ribosomal protein s21

ribosomal protein s7

ribosomal protein s8

riken cdna 2310046k01

ring finger protein 11

ring finger protein 213

rna binding motif protein 28

rna binding motif protein 5

rna polymerase ii-associated protein 3

rna terminal phosphate cyclase-like 1

rna-binding protein pno1

ryanodine receptor skeletal muscle

s100 calcium binding protein a1

s100 calcium binding protein beta variant 1

s238b_danre ame: full=solute carrier family 25 member 38-b

s-adenosylmethionine synthetase isoform type-2

salmon calcitonin-ia precursor

samd9l protein

sarcoplasmic endoplasmic reticulum calcium atpase 2

scinderin like a

sec23 homolog b ( cerevisiae)

sec61 alpha like 1

serine cysteine proteinase inhibitor

serine protease 27 precursor

serine threonine kinase cdc2

serine threonine protein kinase pim-3

serine threonine-protein kinase pim-3

serine threonine-protein kinase sgk1

serum glucocorticoid regulated kinase

serum glucocorticoid regulated kinase 1

serum lectin isoform 3

set and mynd domain containing 3

set domain-containing protein 6

sh3-domain binding protein 5 (btk-associated)

shc sh2-domain binding protein 1

shc-transforming protein 2

short chain dehydrogenase reductase family protein

si:ch211- protein

si:dkey- isoform 1

si:dkey- protein

sialidase

signal transducer and activator of transcription 1

signal transducer and activator of transcription 3 isoform 2

sjchgc05390 protein

sjchgc09533 protein

skin mucus lectin

slc22a17 protein

slowmo homolog 2

small inducible cytokine a4

smc hinge domain containing 1

smc2 protein

smc4 protein

sodium potassium-transporting atpase subunit beta-233

sodium-coupled neutral amino acid transporter 2

solute carrier family 10 (sodium bile acid cotransporter family) member 1

solute carrier family 10 (sodium bile acid cotransporter family) member 7

solute carrier family 15 (oligopeptide transporter) member 1

solute carrier family 2 (facilitated glucose fructose transporter) member 5

solute carrier family 2 (facilitated glucose transporter) member 6

solute carrier family 22 (extraneuronal monoamine transporter) member 3

solute carrier family 22 member 1

solute carrier family 22 member 2

solute carrier family 22 member 7

solute carrier family 25 (mitochondrial carrier phosphate carrier) member 25

solute carrier family 25 (mitochondrial carrier: glutamate) member 22

solute carrier family 25 member 33

solute carrier family 34 (sodium phosphate) member 2

solute carrier family 39 (zinc transporter) member 8

solute carrier family 7 (cationic amino acid y+ system) member 1

solute carrier family member 1

solute carrier family member 17-like

solute carrier family member 2

solute carrier family member 28

solute carrier family member 29

solute carrier family member 33

solute carrier family member 38

solute carrier family member 43

solute carrier family member 4-like

solute carrier family member 6 (electroneutral potassium-chloride cotransporter 3) (k-cl cotransporter 3) isoform 1

solute carrier family member 9b

sorting nexin 4

sorting nexin-10

spastic ataxia of charlevoix-saguenay

sperm associated antigen 5

spermatogenesis associated 5

spla ryanodine receptor domain and socs box containing 1

splicing factor u2af 65 kda

splicing factor u2af large

squalene epoxidase

src family associated phosphoprotein 1

src-like-adaptor 2

stathmin

stearoyl- desaturase

sterol o-acyltransferase 1

sterol-c4-methyl oxidase-like

stress 70 protein microsome- 60kda

structural maintenance of chromosomes 2

structure protein nsp5b3b

stxa_synve ame: full=neoverrucotoxin subunit alpha short= subunit alpha

stxb_synve ame: full=neoverrucotoxin subunit beta short= subunit beta

subfamily member 1

subfamily member 9

subfamily member a1-like

subtilase family serine

succinate dehydrogenase iron-protein subunit

sucrase-isomaltase (alpha-glucosidase)

sulfite oxidase

suppressor of cytokine signaling 1

suppressor of cytokine signaling 2

suppressor of cytokine signaling 8

suppressor of zeste 12 homolog

surfeit 6

sushi domain containing 2

sushi domain containing 2-like

swim zinc finger domain-containing protein

syntabulin isoform b

syntabulin isoform d

t cell receptor alpha chain

tap binding

tap binding protein

targeting protein for xklp2

t-cell activation protein phosphatase 2c

t-cell receptor alpha

t-cell surface antigen cd2 precursor

t-complex protein 1 subunit alpha

tena thi-4 family

terf1 -interacting nuclear factor 2

tetratricopeptide repeat domain 25

thap domain-containing protein 4

thiosulfate sulfurtransferase kat

thrombospondin 4

thymidine kinase 1

thymidylate kinase

thymidylate kinase family lps-inducible member

thyroid hormone receptor associated protein 3

tissue factor pathway inhibitor 2 precursor

toll-like receptor 5

toll-like receptor 5s

tpa: tpa_exp: replicase helicase endonuclease

tpa: tpa_inf: eukaryotic translation elongation factor 1a

tpa: tpa_inf: fam20c2

tpa: tpa_inf: fam20c3

tpa: tpa_inf: fam20c4

tpa: tpa_inf: rtn1

tpa_exp: transposase

tpa-induced transmembrane protein homolog

traf and tnf receptor associated protein

traf-type zinc finger domain-containing protein 1

transcription elongation factor a 3

transcription factor cp2-like protein 1

transcription factor lbp-9

transcription factor mitochondrial

transcriptional regulator myc-2

translation elongation factor 1-alpha

translation initiation factor

translation initiation factor eif-2b subunit epsilon

transmembrane 4 l6 family member 4

transmembrane 7 superfamily member 2

transmembrane protein 150c

transmembrane protein 16b

transmembrane protein 38b

transmembrane protein 90b-like

transmembrane protein c9orf91 homolog

transport protein sec61 subunit alpha

transport protein sec61 subunit gamma

transporter 2 atp-binding cassette sub-family b

transposable element tc1 transposase

transposable element tcb1 transposase

transposase

transposase [Salmo salar]

transposase domain-containing protein

transposase homolog

tribbles homolog 1

tripartite motif-containing 25

tripartite motif-containing 8

trna methyltransferase 61 homolog a

tropomyosin-1 alpha chain

trp3_pseam ame: full=trypsinogen-like protein 3 flags: precursor

truncated poly

trypsinogen 2

trypsinogen ii precursor

ttk protein kinase

ttrap protein

tubulin alpha chain - sea urchin (paracentrotus lividus)

tudor domain containing 1

tuftelin 1

tumor necrosis factor receptor associated factor 2

tumor necrosis factor receptor superfamily member 11b precursor

tumor necrosis factor receptor superfamily member 14 precursor

tumor protein p53 inducible protein 3

tumor-associated calcium signal transducer 2 precursor

type i cytoskeletal 13

type i cytoskeletal 18

type i enveloping like

type ii

type ii antifreeze protein 1

type ii cax cation proton exchanger

type ii keratin

type ii keratin e3

type iii iodothyronine deiodinase

tyrosine aminotransferase

tyrosine phosphatase type iva 2

ubiq_strpu ame: full=ubiquitin

ubiquitin c

ubiquitin carboxyl-terminal hydrolase domain containing protein

ubiquitin specific peptidase 18

ubiquitin specific peptidase 35

ubiquitin specific protease 18

ubiquitin-activating enzyme 5

ubiquitin-activating enzyme e1

ubiquitin-conjugating enzyme e2 c

ubiquitin-conjugating enzyme e2 e1

ubiquitin-conjugating enzyme family protein

ubiquitin-like protein 1

ubiquitin-like protein precursor

unc-51 like kinase 2 ( elegans)

unknown protein [Siniperca chuatsi]

unnamed protein product [Tetraodon nigroviridis]

uridine phosphorylase 2

uridine-cytidine kinase 2

vacuolar sorting atpase

very large inducible gtpase-1-like

v-fos fbj murine osteosarcoma viral oncogene homolog

vhsv-induced protein

vhsv-induced protein-10

viral myc transforming protein

vit1_funhe ame: full=vitellogenin-1 ame: full=vitellogenin i short=vtg i contains: ame: full=lipovitellin-1 short=lv1 contains: ame: full=phosvitin short=pv contains: ame: full=lipovitellin-2 short=lv2 flags: precursor

vitellogenin a

vitellogenin b

vitellogenin c

von willebrand factor a domain containing 5a-like

v-rel reticuloendotheliosis viral oncogene homolog a

wd repeat domain 19

wd repeat domain 74

whey acidic protein precursor

wolf-hirschhorn syndrome candidate 2

x-box binding protein 1

XNop56 [Oreochromis mossambicus]

xtp3-transactivated gene a protein homolog

yrinic acid -diamide mitochondrial precursor

zeta-chain associated protein kinase 70kda

zgc:110749 protein

zgc:162544 protein

zgc:173425 protein

zgc:174680 protein

zgc:175135 protein

zgc:192870 protein

zgc:55406 protein

zgc:56053 protein

zgc:73336 protein

zinc an1-type domain 5

zinc an1-type domain 5a

zinc finger

zinc finger (c3hc4 ring finger) protein

zinc finger (c3hc4 type) fha domain-containing protein

zinc finger and btb domain containing isoform cra_b

zinc finger ccch type containing 12a

zinc finger partial

zinc finger protein

zinc finger protein 300-like

zinc finger protein 313

zinc finger protein isoform cra_a

zinc hit type 6

zinc mynd-type containing 19

zinc nfx1-type containing 1

zygotic dna replication licensing factor mcm6-b

Table S3. Site history of hypoxia.

| Site | Abbreviation | Record of Hypoxia | Reported by |
| --- | --- | --- | --- |
| **Port Aransas / Aransas Bay, TX** | **PA** | **First in 1988, recurring 1988-1996** | **Ritter and Montagna (1999);**  **Engle et al. (1999)** |
| Port O’Conner / Matagorda Bay, TX, mouth of the bay | PO | No record, reported as an undegraded reference | Engle et al. (1994) |
| Lake Charles / Lake Calcasieu, LA | LC | No record, reported as an undegraded reference | Engle et al. (1994) |
| **Terrebonne-Bartaria / Leeville, LA** | **LV** | **Offshore zone to mouth of bay, One year measurement out of four year study** | **Rabalais and Turner (2001);**  **Engle et al. (1999)** |
| Weeks Bay, AL / Mobile Bay interior | WB | Long record of hypoxia, dating to 1820’s | May (1973); US EPA, Engle et al. (1994); Engle et al. (1999). |
| **Mobile Bay-Dauphin Island AL / Pass Aux Heron** | **DA** | **No record found** |  |

Table S4 - RIN values for RNA samples

| **Treatment** | **Sample** | **RIN value** |
| --- | --- | --- |
| Unexposed | U1 | 8.4 |
|  | U2 | 8.3 |
| Exposed | E1 | 9.7 |
|  | E2 | 9.2 |
|  | E3 | 9.4 |
